# B7 homolog 3-targeted CAR-T cells secreting EGFR T-cell engagers for improved control of glioblastoma progression

**DOI:** 10.1186/s43556-026-00492-7

**Published:** 2026-06-16

**Authors:** Zongliang Zhang, Nian Yang, Hui Zeng, Yongdong Chen, Huaqing Lu, Long Xu, Zeng Wang, Guoqing Wang, Liangxue Zhou, Aiping Tong

**Affiliations:** 1 State Key Laboratory of Biotherapy and Cancer Center, Research Unit of Gene and Immunotherapy, Chinese Academy of Medical Sciences, Collaborative Innovation Center of Biotherapy, West China Hospital, Sichuan University, Chengdu Sichuan Province, 610041 China; 2 Department of Neurosurgery, Mianyang Central Hospital, Mianyang, 621000 Sichuan China; 3 Department of Ophthalmology, West China Hospital, Sichuan University, West China Medical School, Chengdu, 610041 Sichuan China; 4 Department of Neurosurgery, West China Hospital, West China Medical School, Sichuan University, Chengdu, 610041 Sichuan China; 5 Frontiers Medical Center, Tianfu Jincheng Laboratory, Chengdu, 610212 China

**Keywords:** GBM, CAR-T, Bispecific T-cell engager, B7-H3, EGFR

## Abstract

Glioblastoma (GBM) is the most aggressive primary malignant brain tumor, and while chimeric antigen receptor-T (CAR-T) cell therapy has shown promise, its efficacy remains limited by antigen heterogeneity and immune escape. Here, we investigated the expression of B7 homolog 3 (B7-H3), epidermal growth factor receptor (EGFR), and interleukin-13 receptor alpha 2 (IL-13RA2) in GBM tissues and cell lines. Although all three antigens were highly expressed, sustained exposure to B7-H3 CAR-T cells led to significant B7-H3 downregulation but concurrent EGFR upregulation, revealing a potential immune escape mechanism. To address this heterogeneity, we engineered T cells to express an anti-B7-H3 CAR and secrete an EGFR-targeting bispecific T-cell engager (EGFR-BsTe). These B7-H3-CAR-T-EGFR-BsTe cells exerted dual functionality: direct B7-H3-dependent cytotoxicity and recruitment of unmodified T cells via secreted EGFR-BsTe to eliminate EGFR-expressing tumor cells. Notably, EGFR-BsTe secretion promoted CAR-T cell proliferation and effector differentiation. In orthotopic GBM xenograft models, including mixed tumors with heterogeneous antigen expression, B7-H3-CAR-T-EGFR-BsTe cells demonstrated superior antitumor activity and prolonged survival compared to conventional B7-H3 CAR-T cells. Quantitative analysis revealed that EGFR-BsTe secretion abrogated EGFR upregulation and enhanced B7-H3 downregulation in a target-dependent manner; however, efficacy was diminished when the CAR-Target (B7-H3) was absent on a substantial fraction of tumor cells. Our findings suggest that arming B7-H3 CAR-T cells with EGFR-targeting bispecific engagers represents a promising strategy to overcome antigen heterogeneity and improve therapeutic outcomes for GBM patients.

**Supplementary Information:**

The online version contains supplementary material available at 10.1186/s43556-026-00492-7.

## Introduction

Glioblastoma (GBM), classified as a World Health Organization (WHO) grade IV glioma, represents the most common and aggressive primary malignant brain tumor in adults, with a median overall survival of less than 15–24 months and a 5-year survival rate below 10% [1]. The current standard-of-care—maximal safe surgical resection followed by radiotherapy and temozolomide chemotherapy—remains non-curative [2]. Tumor recurrence is virtually inevitable due to the infiltrative nature of GBM, its intrinsic resistance mechanisms, and profound molecular heterogeneity. Thus, the development of innovative therapeutic strategies for this devastating disease is an urgent priority.

CAR-T cell therapy has achieved remarkable success in hematologic malignancies, yet its efficacy in solid tumors, including GBM, remains limited [3]. Key obstacles include the immunosuppressive tumor microenvironment (TME), intrinsic antigen heterogeneity, and the capacity of tumors to evade CAR-T cell pressure through target antigen downregulation [4]. Indeed, clinical trials of CAR-T cells targeting IL13RA2 or EGFR deletion variant III (EGFRvIII) in recurrent GBM have demonstrated objective responses but also revealed significant antigen loss as a mechanism of acquired resistance [5, 6]. To overcome these barriers, next-generation strategies have focused on engineering CAR-T cells with multivalent targeting capabilities, such as bispecific CARs or CARs engineered to locally deliver additional therapeutic payloads (e.g., bispecific T-cell engagers) to remodel the TME and engage bystander immune cells [7, 8].

EGFR is a compelling therapeutic target in GBM, with EGFR amplification and/or mutation occurring in over 50% of cases [9, 10]. The tumor-specific mutant EGFRvIII, present in approximately 30–40% of GBMs, has been extensively pursued using monoclonal antibodies (e.g., cetuximab) and CAR-T cells, with early-phase trials demonstrating tumor regressions [11, 12]. However, targeting EGFR/EGFRvIII presents substantial challenges. First, basal EGFR expression in normal tissues (e.g., skin and gastrointestinal tract) poses risks of "on-target, off-tumor" toxicities [13]. Second, intratumoral heterogeneity and compensatory pathway activation frequently drive resistance. Consequently, there is a critical need for additional tumor-restricted antigens and localized therapeutic approaches that can safely and effectively target EGFR while mitigating resistance.

B7-H3 has emerged as a particularly attractive immunotherapeutic target for GBM. B7-H3 exists as two isoforms (2IgB7-H3 and 4IgB7-H3) and is differentially overexpressed on GBM tumors with minimal expression in vital normal tissues [14–16]. Our group and others have validated the preclinical efficacy of B7-H3-targeting CAR-T cells across multiple solid tumor models, leading to numerous active clinical trials in brain, ovarian, and liver cancers [17–24]. However, accumulating evidence shows that prolonged CAR-T cell pressure may induce B7-H3 loss on tumor cells, ultimately resulting in relapse. While bispecific CARs targeting B7-H3 and other GBM-associated antigens (e.g., IL13RA2) have shown feasibility, alternative combinatorial strategies are needed to address the profound heterogeneity of GBM [5, 8, 25]. Importantly, no study to date has investigated the combined therapeutic targeting of B7-H3 and EGFR in GBM.

Here, we postulated that engineering T cells to simultaneously express a B7-H3-directed CAR and secrete an EGFR/CD3 bispecific T-cell engager (EGFR-BsTe) could overcome both antigen heterogeneity and the inherent drawbacks of EGFR-targeted therapy. We found that these engineered cells—termed B7-H3-CAR-T-EGFR-BsTe—exert dual antitumor activity: direct, B7-H3-dependent cytotoxicity and recruitment of bystander T-cells via secreted EGFR-BsTe. Importantly, EGFR-BsTe secretion enhanced CAR-T cell proliferation and effector differentiation. In orthotopic GBM xenograft models, local intratumoral delivery of EGFR-BsTe by CAR-T cells improved immune infiltration and demonstrated superior antitumor efficacy compared to conventional B7-H3 CAR-T cells. This localized approach enabled effective targeting of EGFR while mitigating the risks of systemic on-target/off-tumor toxicity. Our findings present a translatable strategy to overcome antigen heterogeneity and enhance CAR-T cell therapy for GBM.

## Results

### Persistent B7-H3 CAR-T cell pressure reduces B7-H3 expression but upregulates EGFR in GBM cells

**Fig. 1:**
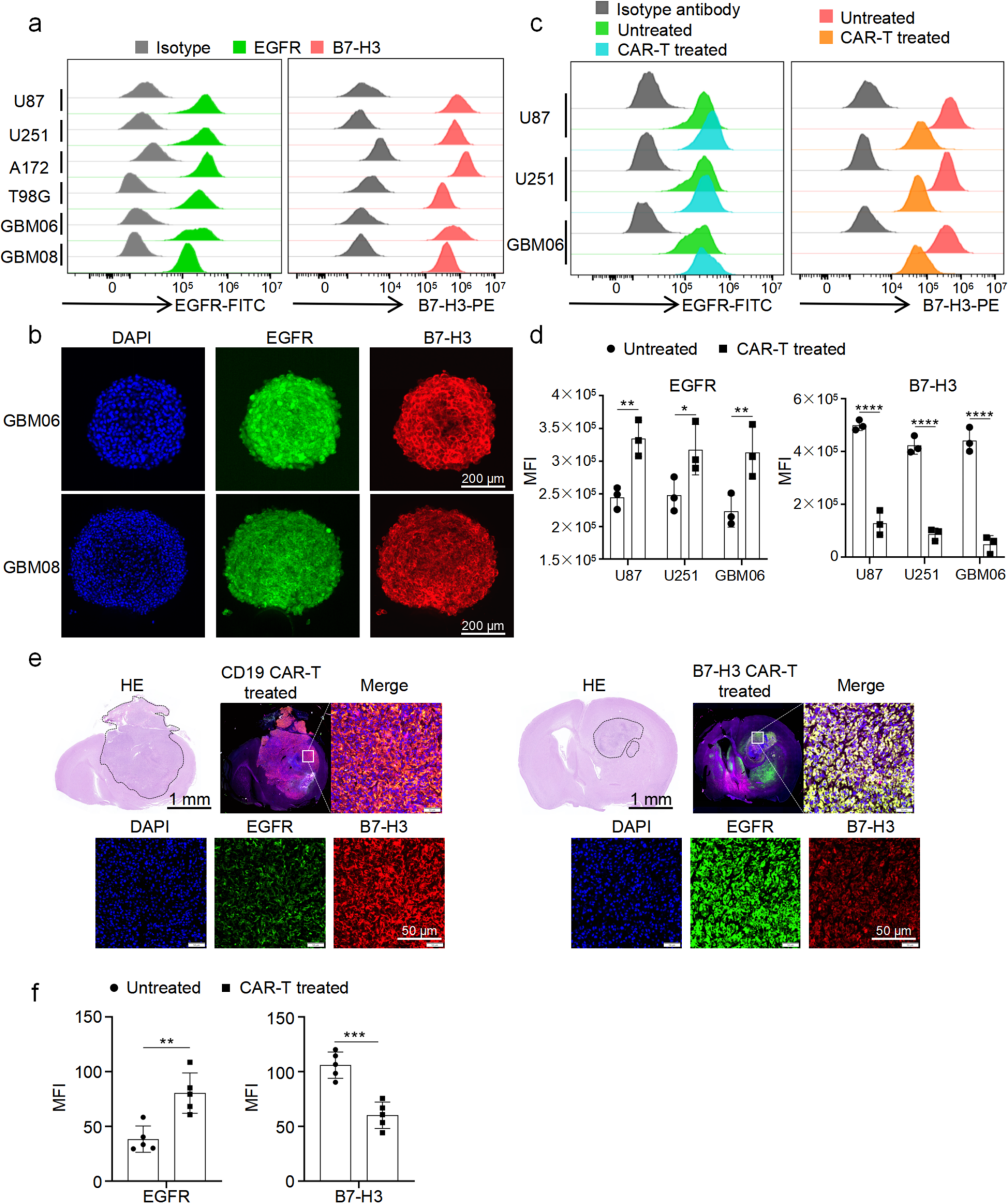
B7-H3 is downregulated and EGFR is upregulated in tumor cells after treatment with B7-H3 CAR-T cells. **a** Surface expression of EGFR and B7‑H3 was evaluated by flow cytometry in four glioma cell lines (U87, U251, A172, T98G) and in two patient‑derived primary GBM cultures (GBM06 and GBM08). **b** Sample immunofluorescence images of EGFR and B7-H3 in organoids derived from GBM06 and GBM08 patients. Scale bars, 200 μm. **c** Representative flow cytometry histograms showing EGFR and B7-H3 expression on residual GBM cells after co-incubation with B7-H3 CAR-T cells, compared to untreated controls. **d** The mean fluorescence intensity (MFI) was analyzed between the untreated group and the B7-H3 CAR-T treated group. Means and SDs are shown (*n* = 3); a two-tailed unpaired Student’s t-test. *P < 0.05; **P < 0.01; ****P < 0.0001; ns, not significant. **e** Representative tumor-burdened brains stained with H&E. Scale bars: 1 mm. The areas outlined with dashed lines indicate the presence of tumors. Coronal sections of the cerebrum from mice treated with CD19 CAR-T or B7-H3 CAR-T cells. Multiplex immunohistochemistry of the coronal brain sections, staining for DAPI, EGFR, and B7-H3. The white boxes highlight regions magnified below. Scale bars: 50 µm. **f** The MFI of EGFR and B7-H3 was compared between the CD19 CAR-T-treated group and the B7-H3 CAR-T-treated group. Means and SDs are shown (n = 5); Data were analyzed by unpaired t-test. **P < 0.01; ***P < 0.001; ns, not significant

To investigate the baseline expression of immunotherapeutic targets in GBM and assess their dynamics under CAR-T cell selection pressure, we first characterized B7-H3, EGFR, and IL13RA2—three common CAR-T targets in glioma—across a panel of GBM models. Flow cytometry confirmed consistent cell surface expression of B7-H3, EGFR, and IL13RA2 across four GBM cell lines, two primary GBM cultures, and two patient-derived organoids (Fig. 1a, b; Fig. S1a–c). These results are consistent with previous reports [9, 26].

When incubated with target cells for 24 h at an effector‑to‑target (E:T) ratio of 1:1, B7‑H3 CAR‑T cells, unlike CD19 CAR‑T controls, demonstrated potent tumor cell killing and significant T‑cell activation, measured through IFN‑γ release (Fig. S2a and S2b). However, when residual tumor cells were subjected to sustained B7-H3 CAR-T cell pressure for three days at the same E:T ratio, a population of surviving cells persisted (Fig. 1c, d, Fig. S3a and S2b). Characterization revealed that these cells had significantly lower surface B7-H3, maintained IL13RA2, and upregulated EGFR. This same antigenic change was observed in vivo. In mice bearing GBM06 xenografts, tumors that received B7-H3 CAR-T cells showed reduced B7-H3 levels, unchanged IL13RA2, and elevated EGFR expression relative to tumors treated with control CD19 CAR-T cells (Fig. 1e, f, Fig. S3c and S3d). Thus, persistent B7‑H3 CAR‑T pressure selects for variants with decreased B7‑H3 but increased EGFR, supporting combinatorial targeting of both antigens.

### High co-expression of B7-H3 and EGFR correlates with poor prognosis in GBM patients

**Fig. 2:**
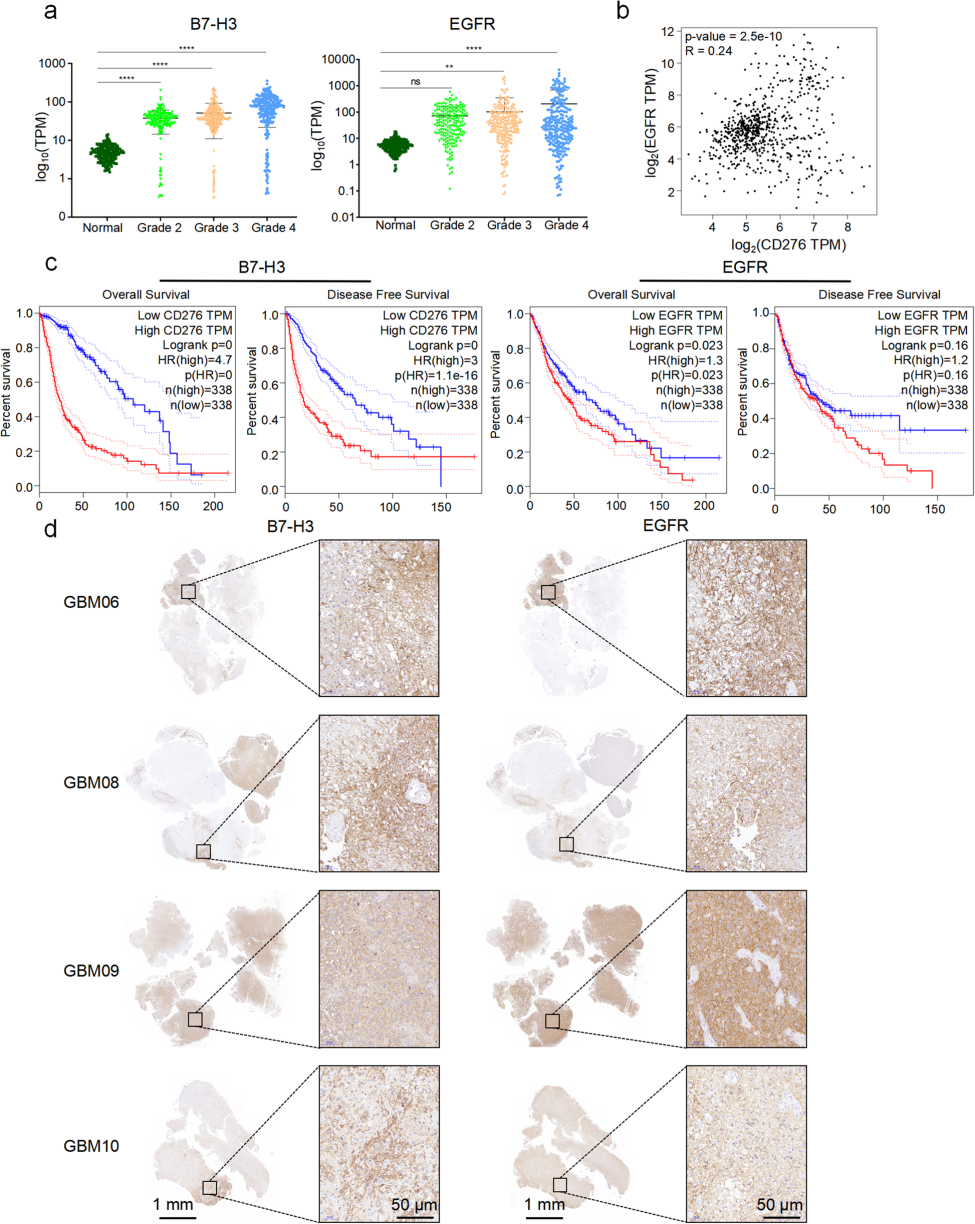
B7-H3 and EGFR co-express in GBM and are correlated with the survival of GBM patients. **a** The mRNA expression levels of B7-H3 and EGFR were analyzed in normal brain tissues and glioma samples using data from the GTEx, and CGGA databases. The normal brain tissue data was sourced from the GTEx database, with n = 181 samples. The CGGA database contributed data for 188 Grade 2 glioma samples, 254 Grade 3 glioma samples, and 247 Grade 4 glioma samples. Values are presented as mean ± SD; one-way ANOVA with Tukey’s test; **P < 0.01; ****P < 0.0001; ns, not significant. **b** Using the GEPIA database, we analyzed the co-expression of B7-H3 and EGFR in glioma specimens. The heatmap (or dot plot) shows the correlation between B7-H3 and EGFR transcript levels. **c** Overall survival (OS) and disease‑free survival (DFS) were analyzed by the Kaplan‑Meier method in glioma patients categorized according to high or low expression levels of B7‑H3 and EGFR. Data were analyzed using the GEPIA database. High expression of both B7-H3 and EGFR correlated with significantly worse OS and DFS. **d** Representative IHC images of B7-H3 and EGFR expression in four freshly resected patient-derived GBM tissues. Brown staining indicates positive expression. Scale bars, 50 µm

Given our observation that EGFR is upregulated under B7-H3 CAR-T pressure, we next evaluated the clinical relevance and co-expression patterns of these two antigens in glioma patients. As shown in Fig. 2a, B7-H3 was highly expressed in both glioblastoma (GBM, WHO grade 4) and low-grade glioma (LGG, WHO grade 1–2), whereas *EGFR* expression was elevated specifically in GBM but remained relatively low in LGG. GEPIA analysis confirmed significant co‑expression in glioma specimens (Fig. 2b). To assess the prognostic significance of these antigens, we performed Kaplan–Meier survival analyses. Patients whose tumors showed high expression of B7‑H3 or EGFR had significantly worse overall and disease‑free survival than those with low levels (Fig. 2c), implying that both antigens are linked to poor prognosis. Concurrently, the protein expression patterns of B7-H3 and EGFR were assessed in four fresh tumor tissues from GBM patients using immunohistochemistry (IHC) (Fig. 2d). The negative control stain was shown in Fig. S4a. As anticipated, all four GBM specimens exhibited strong expression patterns for both B7-H3 and EGFR. Furthermore, it was observed that the GBM specimens had minimal T-cell infiltration (Fig. S4b), highlighting the immunosuppressive nature of the GBM microenvironment. These data provide a strong rationale for engineering T-cells to simultaneously target both B7-H3 and EGFR as a therapeutic strategy for GBM.

**Fig. 3:**
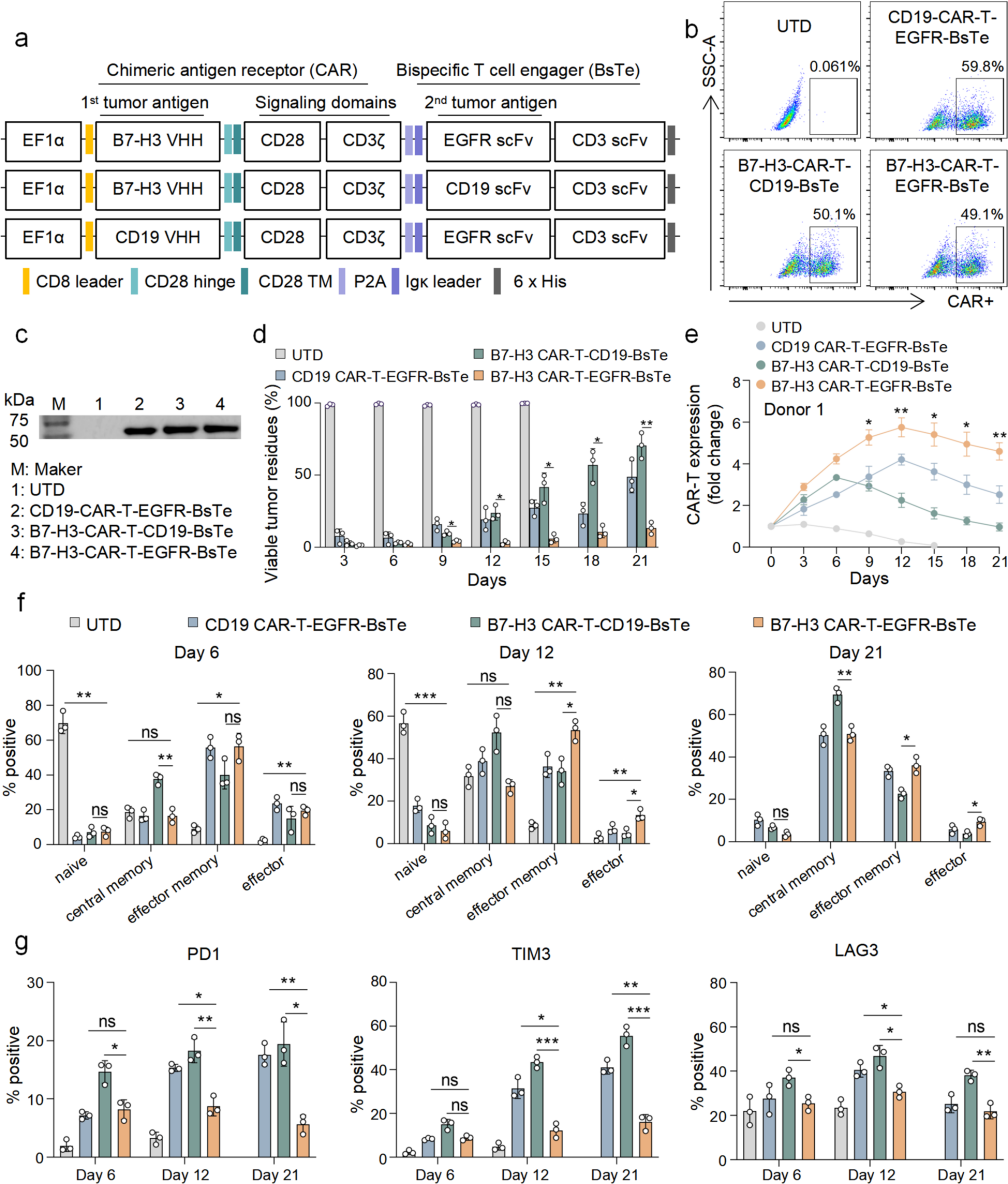
Bispecific T-cell engagers (BsTes) are secreted by CAR-T cells, enhancing CAR-T cells proliferation and differentiation activation. **a** Schematic of the transgene cassettes for two BsTe‑secreting, B7‑H3‑targeted CAR constructs: B7‑H3‑CAR‑T‑EGFR‑BsTe (secretes EGFR‑targeted BsTe) and B7‑H3‑CAR‑T‑CD19‑BsTe (secretes CD19‑targeted BsTe). An additional control construct, CD19‑CAR‑T‑EGFR‑BsTe, was designed to produce EGFR‑BsTe without the B7‑H3 CAR. **b** Flow cytometry showing CAR expression on transduced T cells detected by FITC‑conjugated B7‑H3 or CD19 recombinant protein. **c** Western blot analysis of BsTe proteins in CAR‑T cell culture supernatants using an anti‑His‑tag antibody. Lane assignments: M, marker; 1, untransduced T cells (UTD); 2, CD19‑CAR‑T‑EGFR‑BsTe; 3, B7‑H3‑CAR‑T‑CD19‑BsTe; 4, B7‑H3‑CAR‑T‑EGFR‑BsTe. Samples were run on SDS‑PAGE and transferred for immunoblotting. **d** CAR-T cells were stimulated through repeated coculturing with GBM06 cells over a total of 21 days. The GBM06 cells were refreshed every three days during the serial coculture. Viable tumor residues **d**, CAR-T cell expansion **e**, phenotypes of CAR-T cells (CD45RO and CD62L) **f**, and exhaustion markers of CAR-T cells (TIM-3, PD-1, and LAG-3) **g** were detected in each round of the co-culture assay. Data represent the mean ± SD of triplicate wells; **e** two-way ANOVA with Sidak's multiple comparisons test; (**f** and **g**) two-way ANOVA with Tukey's test; *P < 0.05; **P < 0.01; ***P < 0.001; ns, not significant

### Development and characteristics of B7-H3-CAR-T-EGFR-BsTe Cells, which secrete EGFR-BsTe to enhance proliferation and differentiation activation

To achieve simultaneous dual targeting of B7-H3 and EGFR while also promoting T cell expansion within the GBM TME, we engineered a B7-H3-directed CAR that secretes a BsTe recognizing both EGFR and CD3 (Fig. 3a). The local secretion of EGFR‑targeted BsTes by CAR‑T cells could help avoid the on‑target toxicity that often limits systemically delivered bispecific engagers [26]. Flow cytometric analysis showed that approximately 50% of transduced T cells expressed the B7-H3 CAR, as detected by FITC-labeled recombinant B7-H3 protein, while CD19 CAR expression was detected in a similar percentage of control T cells using FITC-labeled CD19 protein (Fig. 3b). Successful secretion of EGFR‑BsTe or CD19‑BsTe by transduced CAR‑T cells was verified via western blot, which detected the respective proteins at the predicted 55 kDa band (Fig. 3c).

To explore the proliferative capacity, we repeatedly stimulated CAR-T cells from two donors by coculturing them with tumor cells (GBM06 and GBM08). Over the first two stimulation rounds, B7‑H3‑CAR‑T‑EGFR‑BsTe cells expanded more rapidly than B7‑H3‑CAR‑T‑CD19‑BsTe cells, although the difference was not statistically significant (Fig. 3e and Fig. S5a). These findings were corroborated by CFSE assays (Fig. S5b). Meanwhile, both B7-H3-CAR-T-EGFR-BsTe cells and B7-H3-CAR-T-CD19-BsTe cells had obvious tumor-killing activity (Fig. 3d). Following a third round of stimulation, B7‑H3‑CAR‑T‑EGFR‑BsTe cells continued to expand, whereas the control CAR‑T cells showed diminished proliferation. The percentage of viable tumor cells was determined, and the B7-H3-CAR-T-EGFR-BsTe cells had better tumor-killing activity. This pattern persisted through the seventh stimulation. At days 6, 12, 21, exhaustion markers (TIM-3, PD-1, and LAG-3) and memory phenotype (CD45RO, CD62L) were detected by flow-cytometric analysis. Compared to UTD, all CAR‑T cells contained significantly higher proportions of effector memory and effector T cells, and fewer naïve T cells. At day 6, the frequencies of effector memory and effector T cells did not differ significantly between the two CAR‑T cell types; however, significant changes were observed at day 12 and 21 (Fig. 3f and Fig. S6a). Furthermore, exhaustion marker levels were elevated on all CAR‑T cells relative to UTD, but a trend toward lower expression of these markers was observed on both B7‑H3‑CAR‑T‑EGFR‑BsTe and B7‑H3‑CAR‑T‑CD19‑BsTe cells (Fig. 3g and Fig. S6b). Thus, EGFR‑BsTe secretion enhances CAR‑T cell proliferation, persistence, and effector differentiation.

###  B7-H3-CAR-T-EGFR-BsTe cells exhibited better cytotoxic function against GBM cells in vitro

**Fig. 4:**
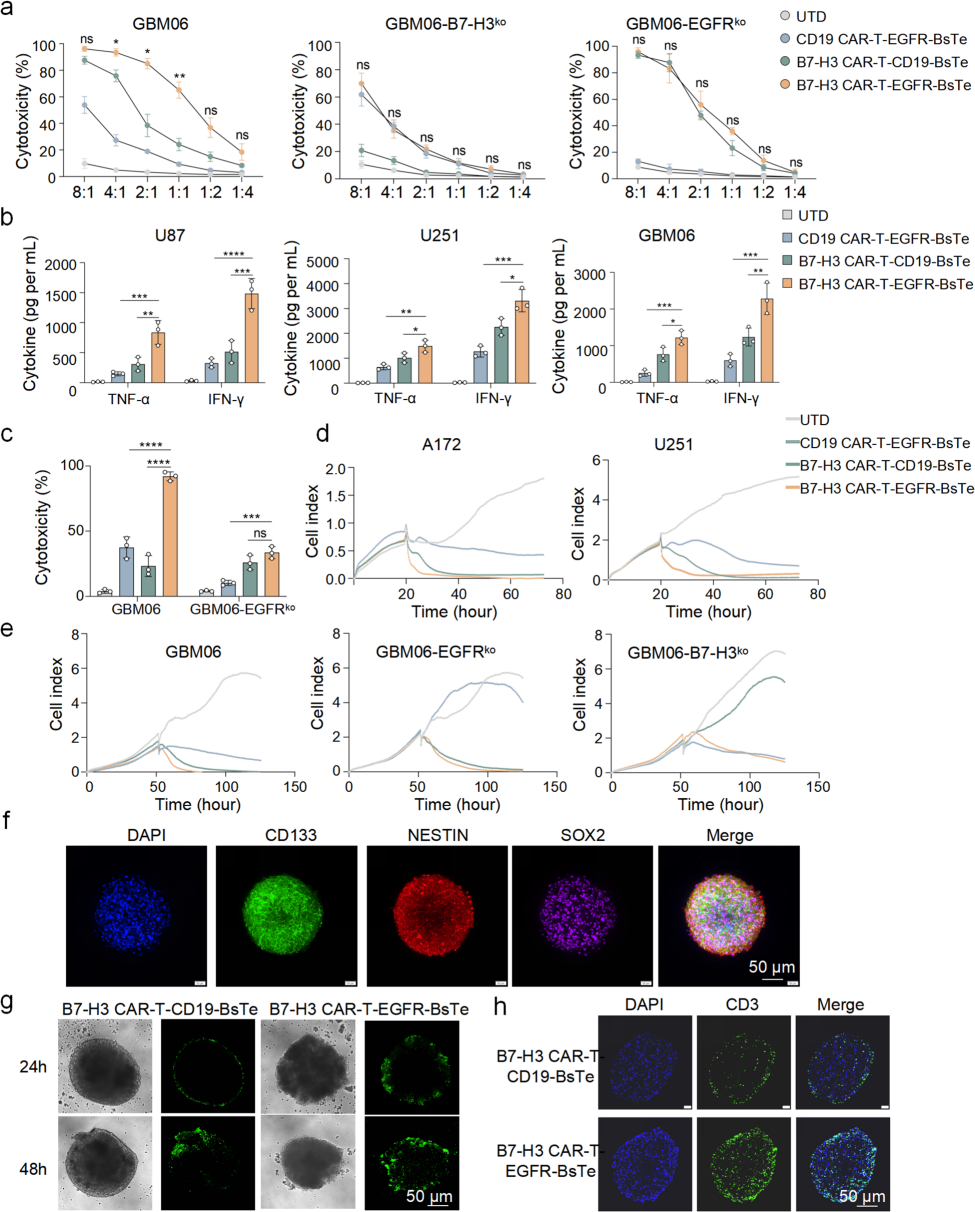
The engineered B7-H3-CAR-T-EGFR-BsTe cells display specific recognition of their target antigens and show strong anti-tumor effects in vitro. **a** Bioluminescence cytotoxicity assay of UTD, CD19-CAR-T-EGFR-BsTe, B7-H3-CAR-T-CD19-BsTe, or B7-H3-CAR-T-EGFR-BsTe cells co-cultured with GBM06, GBM06-B7-H3^ko^, GBM06-EGFR^ko^ cell lines after 18 h at varying effector-to-target (E/T) ratios. The groups of B7-H3-CAR-T-CD19-BsTe and B7-H3-CAR-T-EGFR-BsTe cells were compared and presented as mean ± SD, n = 3 donors. One-way ANOVA with Tukey's test; *P < 0.05; **P < 0.01; ns, not significant. **b** For the measurement of cytokines, UTD, CD19-CAR-T-EGFR-BsTe, B7-H3-CAR-T-CD19-BsTe, or B7-H3-CAR-T-EGFR-BsTe cells were co-cultured with U87, U251, or GBM06 cell lines at an E/T ratio of 1:1 for 18 h. The release of TNF-α and IFN-γ in the culture supernatants of the killing assays was detected by ELISA. Data are mean ± SD; *n* = 3 technical replicates; one-way ANOVA with Tukey's test; *P < 0.05; **P < 0.01; ***P < 0.001; ****P < 0.0001. **c** To further demonstrate the effect of BsTe in CAR-T cell supernatants, we added equal volumes of supernatants from UTD, CD19-CAR-T-EGFR-BsTe, B7-H3-CAR-T-CD19-BsTe, and B7-H3-CAR-T-EGFR-BsTe cells, as well as the same number of primary T cells from various healthy donors co-cultured with GBM06 and GBM06-EGFR^ko^ cell lines. The cytotoxicity mediated by T-cells was analyzed. Data are shown as mean ± SD, n = 3 donors; one-way ANOVA with Tukey's test; ***P < 0.001; ****P < 0.0001; ns, not significant. **d**, **e** Real‑time impedance assay of indicated effector cells (UTD, CD19‑CAR‑T‑EGFR‑BsTe, B7‑H3‑CAR‑T‑CD19‑BsTe, or B7‑H3‑CAR‑T‑EGFR‑BsTe) co‑cultured with A172, U251, GBM06, GBM06‑B7‑H3^ko^, or GBM06‑EGFR^ko^ target cells (E/T = 1:1). **f** Representative immunofluorescence images of GBM06 organoids stained with DAPI, CD133, NESTIN, and SOX2. (Scale bar: 50 μm). **g** The B7-H3-CAR-T-CD19-BsTe and B7-H3-CAR-T-EGFR-BsTe cells were prestained with CFSE dye (green), and subsequently cocultured with GBM06 organoids for 24 h and 48 h, respectively. **h** Following the coculture described in **g**, paraffin sections of the GBM06 organoids were prepared using paraformaldehyde fixation and subsequent paraffin embedding. The paraffin sections were then processed for CD3 (green) immunofluorescence staining. Images from **g** and **h** were captured using a confocal microscope (Olympus Spin), with scale bars measuring 50 μm

To evaluate the antigen-specific cytotoxic activity of B7-H3-CAR-T-EGFR-BsTe cells and determine the contribution of secreted EGFR-BsTe to overall killing efficacy, we performed co-culture experiments with a panel of GBM cell lines, including wild-type GBM06, U87, and U251 cells, as well as B7-H3 knockout (GBM06-B7-H3ko) and EGFR knockout (GBM06-EGFRko) variants. Flow cytometry data confirming efficient knockout in GBM06-B7-H3ko and GBM06-EGFRko cells are presented in Figure S7. Target cells were cultured at fixed numbers while effector cells were added at varying E:T ratios (8:1, 4:1, 2:1, 1:1, 1:2, and 1:4) to generate cytotoxicity curves. Both B7-H3-CAR-T-EGFR-BsTe and B7-H3-CAR-T-CD19-BsTe cells induced substantial, B7-H3-dependent cytotoxicity across all GBM cell lines tested, with comparable killing efficacy observed between the two CAR-T cell populations (Fig. 4a and Fig. S8a). However, analysis of effector molecule release revealed that B7-H3-CAR-T-EGFR-BsTe cells secreted significantly higher levels of Perforin, Granzyme B, TNF-α, and IFN-γ (Fig. 4b and Fig. S8b).

To quantify BsTe secretion and verify its bioactivity, we also measured BsTe concentrations in post-co-culture supernatants using ELISA. Notably, supernatants from B7-H3-CAR-T-EGFR-BsTe cells contained higher levels of EGFR-BsTe compared to those from CD19-CAR-T-EGFR-BsTe control cells (Fig. S8c). Supernatants from these cells contained more EGFR‑BsTe and, when added to bystander T cells, potently killed GBM cells, confirming bioactivity (Fig. 4c). We then established co-cultures of CAR-T cells with the aforementioned GBM cells in real-time cytotoxicity assays at fixed E/T ratios of 2:1. Time-lapse imaging revealed the specific cytolytic effect of B7-H3 CAR against A172, U251, and GBM06 cells; however, B7-H3-CAR-T-EGFR-BsTe cells showed faster tumor cell killing compared with B7-H3-CAR-T-CD19-BsTe cells (Fig. 4d and e). In contrast, less comparable cytotoxicity was observed against GBM06-EGFR^ko^ and GBM06-B7-H3^ko^ GBM cell lines between B7-H3-CAR-T-EGFR-BsTe and B7-H3-CAR-T-CD19-BsTe cells. This indicates that the observed killing is antigen‑specific.

We further evaluated the in vitro antitumor activity of B7-H3-CAR-T-EGFR-BsTe cells using a GBM patient-derived organoid assay to mimic the compactness of GBM. GBM organoids (GBM06 and GBM08) were identified by staining with CD133, NESTIN, and SOX2 (Fig. 4f and Fig. S9a). As depicted in Fig. 4g and Fig. S9b, compared to B7-H3-CAR-T-CD19-BsTe cells, a greater number of B7-H3-CAR-T-EGFR-BsTe cells were deposited at the peripheral rim of tumor spheroid regions and cooperatively attacked the tumor spheroid. Furthermore, CD3 immunofluorescence staining of representative slices from GBM organoids revealed increased infiltration of B7-H3-CAR-T-EGFR-BsTe cells (Fig. 4h, Fig. S9c and S9d). Taken together, B7-H3-CAR-T-EGFR-BsTe cells exhibit B7-H3‑specific cytotoxicity on par with that of B7-H3-CAR-T-CD19-BsTe cells. In addition, they secrete EGFR‑BsTe, which targets EGFR‑positive tumor cells, boosts CAR‑T cell activity, and ultimately produces outstanding antitumor effects.

### B7-H3-CAR-T-EGFR-BsTe cells is efficacious against GBM in mice

**Fig. 5:**
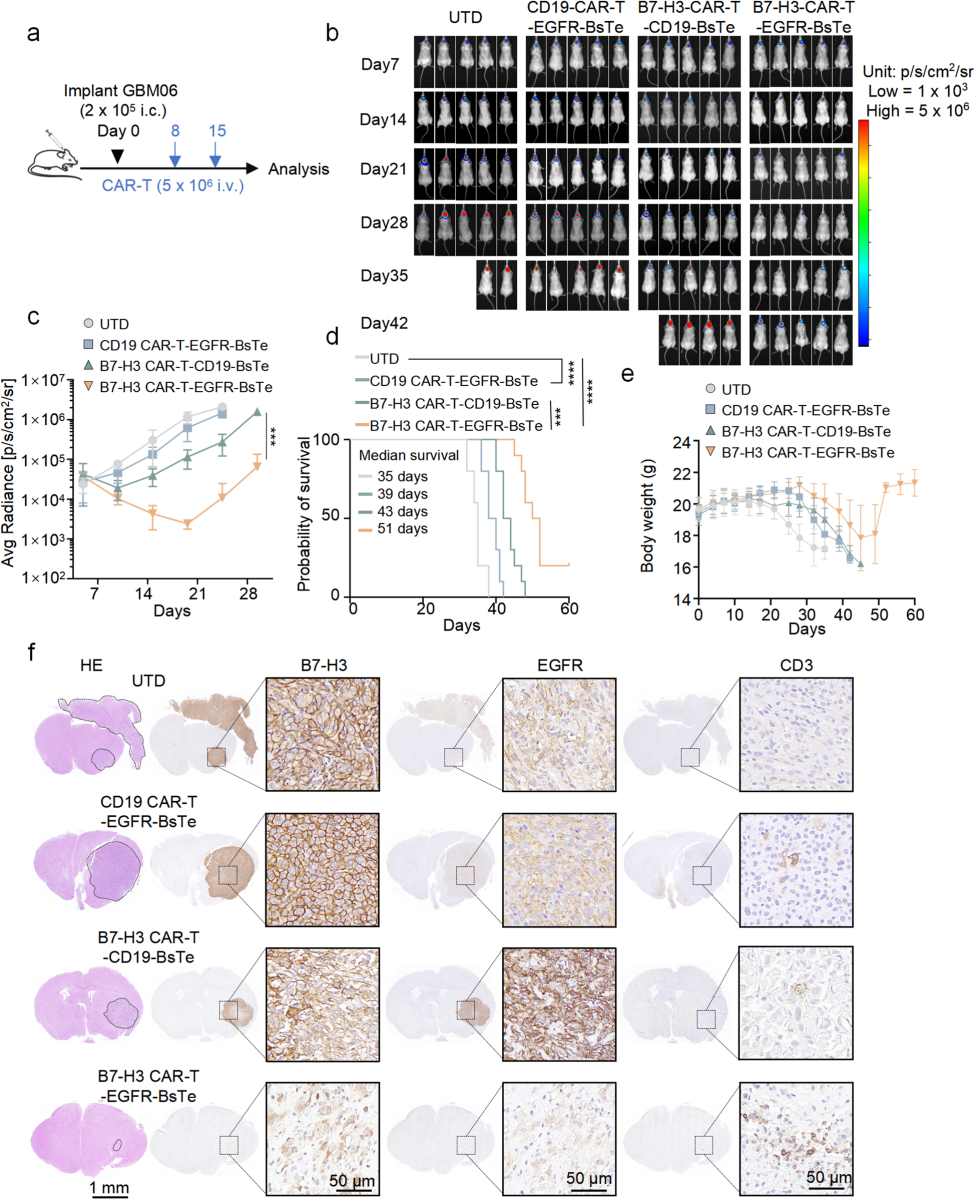
B7-H3-CAR-T-EGFR-BsTe cells showed potential tumor growth suppression in vivo. **a** Schema for glioblastoma xenograft model in NSG mice. CAR-T cells were infused i.v. on day 8 and day 15. Tumor progression monitoring by bioluminescence imaging (BLI) was conducted in a weekly basis. **b** Representative bioluminescence images of tumor signal at indicated time points. n = 5 mice. **c** Tumor growth is represented by average values and quantified weekly tumor signal BLI. Bioluminescence at tumor site is shown as mean ± SD, *n* = 5. One-way ANOVA with Tukey’s multiple comparisons test, *P < 0.05. **d** Kaplan–Meier curve analysis of CAR-T cell treatment outcome in mice, n = 10 (Log-rank test, ***P < 0.001; ****P < 0.0001). **e** The body weight of each experimental mouse was measured twice weekly. **f** Immunohistochemistry confirmed H&E staining of the specimen isolated from experimental mice (scale bar, 1 mm), as well as the expression of B7-H3 and EGFR, and the infiltration of CAR-T cells (CD3) (scale bars, 50 μm)

Intracranial GBM06‑Luc tumors were established in NSG mice, which then received two CAR‑T cell injections via the tail vein on days 8 and 15 (Fig. 5a). Tumor dimensions were tracked using bioluminescence imaging (BLI) (Fig. 5b). The BLI results indicated that B7‑H3‑CAR‑T‑EGFR‑BsTe treatment yielded a marked reduction in tumor burden relative to control groups (Fig. 5c). Notably, Kaplan–Meier survival analysis revealed that mice treated with B7-H3-CAR-T-EGFR-BsTe cells had a prolonged median survival compared to those receiving B7-H3-CAR-T-CD19-BsTe cells or control treatments (Fig. 5d). Body weight measurements showed no significant differences among the four groups throughout the treatment period, except for the final surviving mice in the B7-H3-CAR-T-EGFR-BsTe group (Fig. 5e).

One week following treatment, brains from the experimental mice were harvested for histological analysis. Hematoxylin and Eosin (H&E) staining of representative slices from mice treated with either UTD- or CD19-CAR-T-EGFR-BsTe revealed large, space-occupying tumors. In contrast, mice treated with B7-H3-CAR-T-CD19-BsTe exhibited moderate tumor repression, while those treated with B7-H3-CAR-T-EGFR-BsTe showed dramatically smaller tumors (Fig. 5f). Immunohistochemical staining with antibodies against B7-H3, EGFR, or mouse CD3 indicated the expression of B7-H3 or EGFR targets and the infiltration of CAR-T cells, respectively. Staining for B7-H3 revealed a significant downregulation of B7-H3 in the brains of mice treated with B7-H3-CAR-T-CD19-BsTe and B7-H3-CAR-T-EGFR-BsTe, but not in those treated with UTD or CD19-CAR-T-EGFR-BsTe (Fig. S10). EGFR staining showed a marked upregulation of EGFR in the brains of mice treated with B7-H3-CAR-T-CD19-BsTe, consistent with the description in Fig. 1e; however, no such upregulation was observed in the brains of mice treated with UTD or CD19-CAR-T-EGFR-BsTe, nor in those treated with B7-H3-CAR-T-EGFR-BsTe. CD3 staining indicated a measurable amount of T cells in the brains of mice treated with B7-H3-CAR-T-EGFR-BsTe, fewer T cells in those treated with B7-H3-CAR-T-CD19-BsTe, and a scant number of T cells in the brains of mice treated with UTD or CD19-CAR-T-EGFR-BsTe. Collectively, these data demonstrate that B7-H3-CAR-T-EGFR-BsTe cells exhibit superior antitumor efficacy in an orthotopic GBM model, associated with enhanced tumor regression, prolonged survival, prevention of EGFR-upregulating escape variants, and increased intratumoral T-cell infiltration.

###  B7-H3-CAR-T-EGFR-BsTe cells locally release EGFR-BsTe and enhance intratumor CAR-T cell infiltration in vivo

**Fig. 6:**
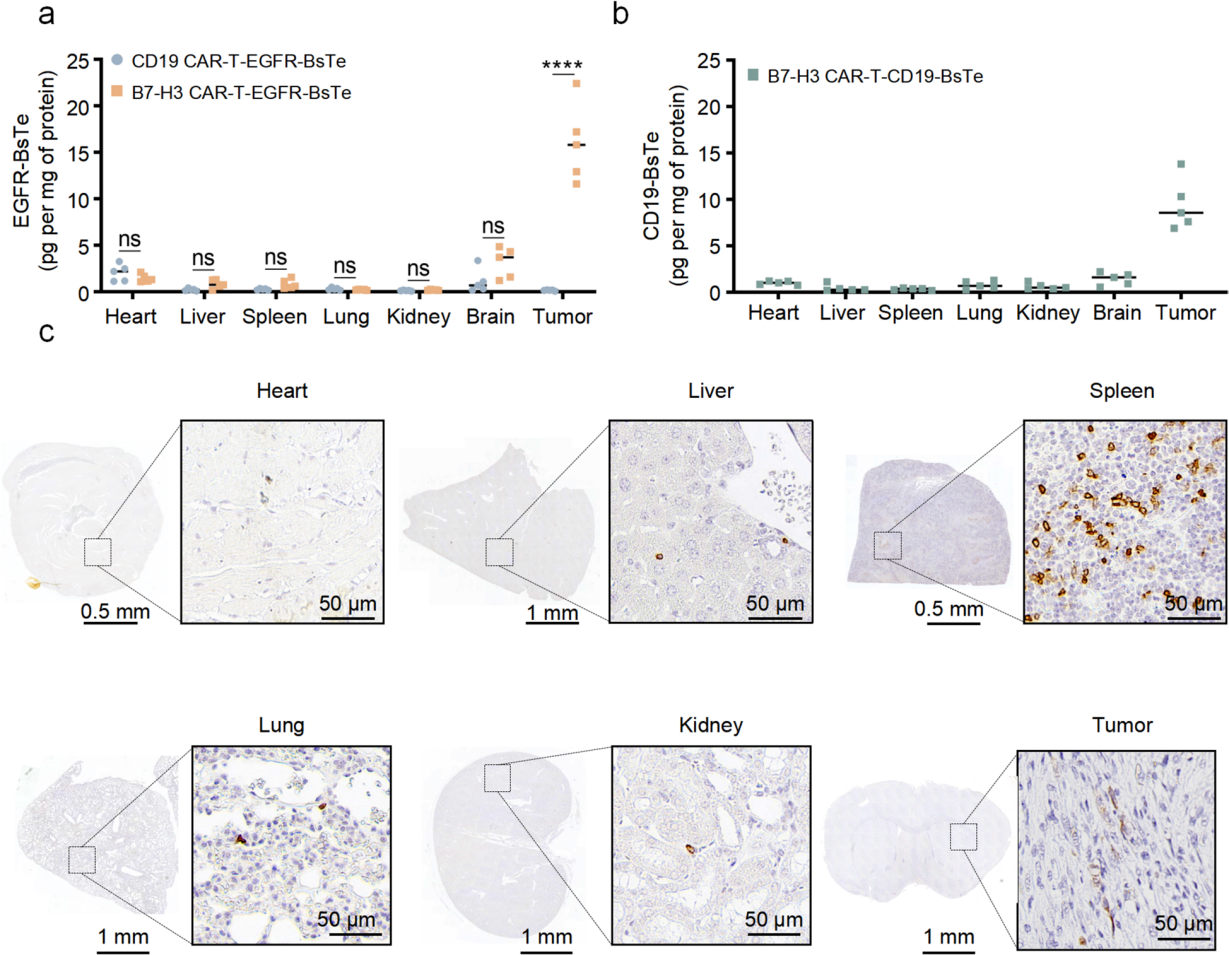
B7-H3-CAR-T-EGFR-BsTe cells locally release EGFR-BsTe, enhancing the accumulation of CAR-T cells in the tumor bed. **a**, **b** Quantification of EGFR-BsTe levels in tumor tissues and indicated normal organs (heart, liver, spleen, lung, kidney, and brain) from mice treated with B7-H3-CAR-T-EGFR-BsTe cells **a** or CD19-CAR-T-EGFR-BsTe cells **b**. BsTe concentrations were measured by ELISA and normalized to total protein content (pg/mg). Data are presented as mean ± SD (n = 5 mice per group). Statistical analysis was performed using two-way ANOVA with Šídák's multiple comparisons test. ****P < 0.0001; ns, not significant. **c** Representative IHC images of CD3 staining in tumor and normal tissues from mice treated with B7-H3-CAR-T-EGFR-BsTe cells, showing human T-cell infiltration. Tissues were harvested 18 days post-tumor implantation (3 days after the second CAR-T cell infusion). Scale bars are indicated

Next, we assessed whether administering B7-H3-CAR-T-EGFR-BsTe cells to mice with GBM06 xenografts would enable tumor‑specific EGFR-BsTe delivery and promote CAR-T cell infiltration into the tumor. Compared to normal mouse tissues, tumor tissues exhibited substantially higher concentrations of EGFR‑BsTe, while no EGFR‑BsTe was detectable in the tumors of CD19‑CAR‑T‑EGFR‑BsTe‑treated mice (Fig. 6a). Similarly, CD19-BsTe was found in the tumors of mice treated with B7-H3-CAR-T-CD19-BsTe cells (Fig. 6b). We also noted human T‑cell infiltration in the mouse spleen (Fig. 6c), which aligns with the known trafficking pattern of human lymphocytes in NSG mice. By contrast, substantial numbers of human CD3 + T cells infiltrated tumors exclusively in the B7-H3-CAR-T-EGFR-BsTe treatment group, validating potent CAR‑T cell recognition of B7-H3‑ and EGFR‑expressing GBM. Together, these findings demonstrate that B7-H3 CAR-mediated tumor recognition enables localized EGFR-BsTe delivery and promotes T‑cell accumulation.

### B7-H3-CAR-T-EGFR-BsTe cells are efficacious against heterogenous tumors

**Fig. 7:**
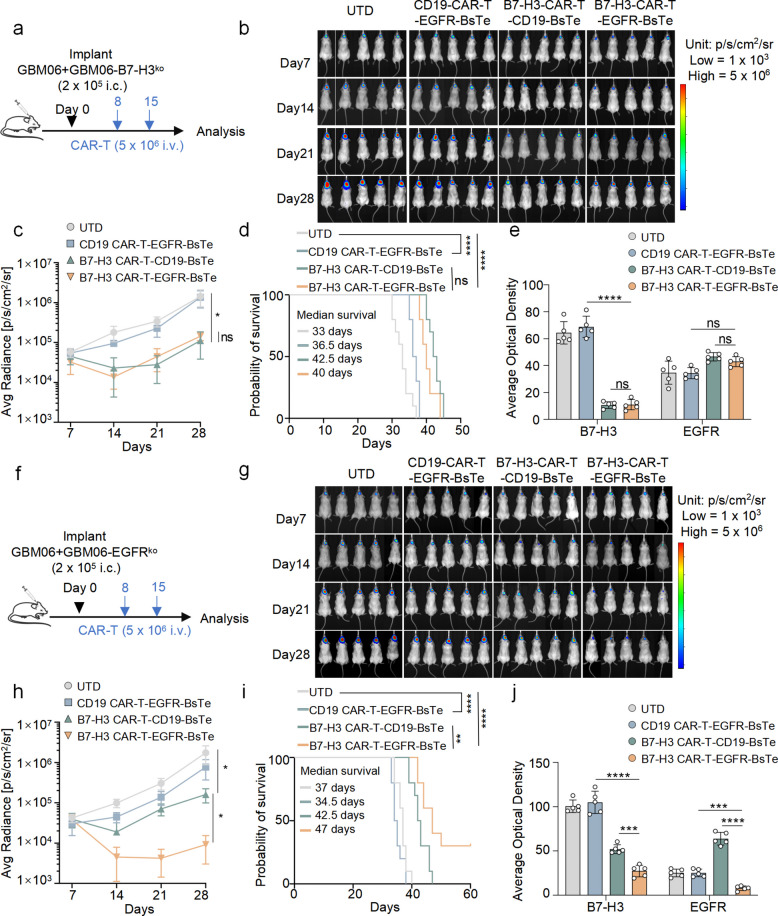
EGFR-BsTe exerts context‑dependent efficacy in orthotopic mixed tumor models. **a** Timeline of mouse models of GBM06 + GBM06-B7-H3^ko^ tumor cells with treatment schedules. On day 0, 1 × 10^5^ GBM06 cells and 1 × 10^5^ GBM06-B7-H3^ko^ cells were intracranially administered into NSG mice. On days 8 and 15, they were intravenously administered with 5 × 10^6^ CAR-T cells or UTD. **b** Representative BLI images of tumors in mice. Tumor burdens were monitored weekly via BLI, and the summarized data are presented in **c**. Each group consisted of five mice (*n* = 5). Values represent mean ± SD, analyzed by one‑way ANOVA with Tukey’s correction; *P < 0.05; ns, not significant. **d** Survival time of GBM06 + GBM06-B7-H3^ko^ tumor-bearing mice was analyzed using the Kaplan–Meier method with the log-rank test (*n* = 10 for each group). ****P < 0.0001; ns, not significant. **e** Quantification of B7-H3 and EGFR expression in tumor sections from the indicated treatment groups. Values represent mean ± SD (*n* = 5). Statistical significance was assessed by one‑way ANOVA with Dunnett’s correction; ****P < 0.0001; ns, non‑significant. **f** Diagram depicting the treatment schedule for the GBM06 + GBM06-EGFR^ko^ tumor model. This model was established by i.c. injection of 1 × 10^5^ GBM06 cells and 1 × 10^5^ GBM06-EGFR^ko^ cells simultaneously. On days 8 and 15, the mice were intravenously injected with 5 × 10^6^ CAR-T cells or an equivalent number of UTD cells. **g** Serial bioluminescence imaging of tumor growth was performed; *n* = 5 mice. **h** Tumor growth is displayed as average values, and data are presented as mean values ± SD and were analyzed by one-way ANOVA with Tukey’s correction. *P < 0.05. **i** Survival based on time to end point was plotted using a Kaplan–Meier curve with the log-rank test. **P < 0.01; ****P < 0.0001. **j** Quantification of B7-H3 and EGFR expression in tumor sections from the GBM06 + GBM06-EGFR^ko^ mixed tumor model. Results are shown as mean ± SD (*n* = 5). Comparisons across groups were performed using one‑way ANOVA and Dunnett’s test; ***P < 0.001, ****P < 0.0001

To assess whether secreted EGFR-BsTe is sufficient to mediate antitumor responses against heterogeneous tumors lacking either B7-H3 or EGFR expression, we established two orthotopic mixed tumor models. NSG mice were implanted with either (1) a 1:1 mixture of GBM06 and GBM06-B7-H3^ko^ cells, or (2) a 1:1 mixture of GBM06 and GBM06-EGFR^ko^ cells (Fig. 7a and e). In the GBM06 + GBM06-B7-H3^ko^ model, both B7-H3 CAR-T cell groups inhibited tumor growth compared to controls. However, B7-H3-CAR-T-EGFR-BsTe cells did not exhibit a significant advantage over B7-H3-CAR-T-CD19-BsTe cells in reducing tumor burden (Fig. 7b, c) or prolonging survival (Fig. 7d). Immunohistochemical analysis revealed significant B7-H3 downregulation in tumors from both B7-H3 CAR-T-treated groups, with no difference between them. EGFR expression was comparable across all groups, and no EGFR upregulation was observed in B7-H3-CAR-T-CD19-BsTe-treated tumors in this mixed model (Fig. 7e and Fig. S11a), likely due to the presence of B7-H3-negative tumor cells altering the selection pressure.

In contrast, in the GBM06 + GBM06-EGFR^ko^ model, mice treated with B7-H3-CAR-T-EGFR-BsTe cells demonstrated a clear advantage in inhibiting tumor burden, similar to the GBM06 model, when compared to those receiving B7-H3-CAR-T-CD19-BsTe cells (Fig. 7f, 7 g and 7 h). Also, overall survival analysis showed a significant difference between mice treated with B7-H3-CAR-T-EGFR-BsTe cells and B7-H3-CAR-T-CD19-BsTe cells (Fig. 7i). Immunohistochemical analysis revealed more pronounced B7-H3 downregulation in tumors from B7-H3-CAR-T-EGFR-BsTe-treated mice compared to those receiving control CAR-T cells (Fig. 7j and Fig. S11b). Notably, while tumors from B7-H3-CAR-T-CD19-BsTe-treated mice exhibited marked EGFR upregulation—consistent with our previous observations—this upregulation was completely abrogated in tumors from B7-H3-CAR-T-EGFR-BsTe-treated mice, indicating that secreted EGFR-BsTe effectively targets EGFR-expressing cells that would otherwise escape. Collectively, these results indicate that the benefit of EGFR‑BsTe secretion depends on the presence of the CAR-Target (B7‑H3) to drive local BsTe production, which then eliminates EGFR‑positive escape variants. These results highlight the mechanism of action wherein CAR engagement drives local BsTe production, which then recruits bystander T cells to eliminate antigen-loss variants in a target-dependent manner.

## Discussion

Two main immunotherapeutic approaches for redirecting T cells against tumors are BsTe therapy and CAR‑T cell therapy. Both have demonstrated potent antitumor activity, but their success in solid tumors like GBM has been limited. In this study, we developed a combined strategy: CAR‑T cells engineered to kill B7‑H3‑positive tumor cells directly via a B7‑H3 CAR, and to simultaneously secrete a BsTe that recognizes a second antigen–EGFR. Our results indicated that this approach achieves superior antitumor efficacy compared to conventional B7-H3 CAR-T cells in both in vitro and in vivo GBM models, including patient-derived organoids and orthotopic xenografts.

Antigen modulation has emerged as a leading mechanism of resistance following CAR-T cell therapy, with antigen loss or escape documented in both hematologic malignancies and solid tumors [27, 28]. In GBM, loss of the targeted antigen was observed after EGFRvIII‑directed CAR‑T cell infusion in roughly 70% of patients [12]. To address this challenge, several groups have developed dual-targeting strategies, including bispecific CARs targeting EGFR/IL13RA2 or B7-H3/IL13RA2 [5, 25, 29, 30]. However, to our knowledge, a combinatorial approach simultaneously targeting B7-H3 and EGFR has not been previously reported. Our study fills this gap by demonstrating that B7-H3 CAR-T cells secreting EGFR-BsTe can effectively prevent and overcome EGFR-upregulating escape variants—a phenomenon we observed under sustained B7-H3 CAR-T cell pressure (Fig. 1e, f). Notably, Innovent Biologics' B7-H3/EGFR bispecific antibody–drug conjugate (IBI3001) recently received IND approval in China [31], underscoring the clinical relevance of this target combination.

A key distinction of our approach from conventional BsTe therapy lies in the mode of delivery. Systemically administered BsTes can recruit and activate T cells effectively but often induce dose-limiting toxicities due to off-tumor T-cell engagement [32–34]. By engineering CAR-T cells to locally secrete EGFR-BsTe within the tumor microenvironment, we achieve targeted delivery that mitigates systemic on-target/off-tumor toxicity. This strategy both corroborates and advances the previous study by Choi et al., in which the authors proved the viability of producing EGFR‑BsTe using EGFRvIII‑specific CAR‑T cells [26]. Our study advances this concept by combining B7-H3 CAR-Targeting with EGFR-BsTe secretion, thereby addressing both B7-H3 loss and EGFR upregulation as complementary escape mechanisms.

It has been previously shown that T cell activation triggered by BsTes lacks co‑stimulatory signaling, which is thought to lead to T cell exhaustion and impaired proliferative capacity [35, 36]. While, we observed that EGFR‑BsTe secretion unexpectedly improved both the phenotype and function of the transduced T cells. Upon repeated in vitro stimulation with GBM06 cells, B7‑H3‑CAR‑T‑EGFR‑BsTe cells exhibited a marked proliferation advantage over their B7‑H3‑CAR‑T‑CD19‑BsTe counterparts. Although all CAR‑T cells showed elevated levels of exhaustion markers (TIM‑3, PD‑1, LAG‑3) relative to UTD, there was a tendency for lower expression of these markers in the EGFR‑BsTe‑secreting group. Moreover, B7‑H3‑CAR‑T‑EGFR‑BsTe cells displayed a more pronounced effector memory and effector T‑cell phenotype. These findings agree with those of Bryan D. Choi et al., who reported that CARs engineered with costimulatory endodomains such as 4‑1BB can mitigate BsTe‑driven T‑cell exhaustion, leading to greater expansion and persistence after repeated antigen stimulation [26]. Accordingly, we propose that CAR engagement indirectly augments local BsTe generation by increasing and maintaining the population of secreting T cells. In turn, BsTes target tumor antigens, which stimulates the proliferation and cytotoxic activity of both CAR-T cells and bystander T cells, thereby creating a virtuous cycle of T-cell expansion.

The clinical implications of our findings are substantial. GBM is characterized by profound molecular heterogeneity and an immunosuppressive tumor microenvironment with limited T-cell infiltration [37]. In freshly resected GBM specimens, we observed minimal CD3 + T-cell presence (Fig. S4b), highlighting the need for strategies that recruit and activate T cells within the tumor. B7-H3-CAR-T-EGFR-BsTe cells address this need by combining direct CAR-mediated cytotoxicity with BsTe-mediated recruitment of endogenous T cells. Furthermore, the target combination we selected—B7-H3 and EGFR—is supported by our analysis of The Chinese Glioma Genome Atlas (CGGA) and GEPIA databases, demonstrating that high co-expression of both antigens correlates with poor prognosis (Fig. 2c). This provides a strong rationale for dual targeting in the patient population most in need of effective therapies.

Several limitations of this study should be acknowledged. First, while our mixed tumor models (GBM06 + GBM06-B7-H3^ko^ and GBM06 + GBM06-EGFR^ko^) provided valuable insights into the mechanism of action in heterogeneous settings, they represent artificial systems with defined knockout populations. In clinical reality, antigen heterogeneity is far more complex, involving variable expression levels, dynamic changes over time, and non-genetic mechanisms of escape [38]. Although we quantified antigen downregulation (as shown in Fig. 6e, 6j and Fig. S11), our data reveal that the efficacy of B7-H3-CAR-T-EGFR-BsTe cells is critically dependent on the presence of the CAR-Target (B7-H3). When a substantial fraction of tumor cells lacked B7-H3, the therapeutic advantage conferred by EGFR-BsTe secretion was lost, as CAR engagement and consequently local BsTe production were impaired. This finding underscores the critical question of whether targeting two antigens is sufficient to achieve durable responses or if a third targeting moiety should be incorporated. Theoretically, adding a third specificity could further reduce the likelihood of antigen escape. However, lentiviral vector cargo capacity (~8–10 kb) poses practical constraints for packaging additional transgenes without compromising viral titer or transduction efficiency. Sequential treatment with CAR-T cells of different specificities is an intriguing alternative, but this approach would double manufacturing costs and treatment complexity, and may not necessarily produce superior outcomes due to potential cross-resistance or overlapping antigen loss. Future studies should explore these possibilities, perhaps leveraging newer vector systems or combinatorial protein engineering approaches.

Second, due to cross-species immune barriers, we were unable to evaluate B7-H3-CAR-T-EGFR-BsTe cells in a syngeneic, immunocompetent murine GBM model. Such models would enable investigation of interactions with endogenous immune cells and assessment of long-term immune memory. While we utilized patient-derived organoids and orthotopic xenografts in NSG mice—which provide valuable human-specific preclinical data—these models lack a functional adaptive immune system and do not fully recapitulate the complexity of the tumor microenvironment [39, 40].

Third, the anti-EGFR scFv used in our BsTe construct is derived from cetuximab and is human-specific, with no cross-reactivity to murine EGFR. Consequently, we could not fully assess the potential on-target/off-tumor toxicity of secreted EGFR-BsTe in our mouse models. This is particularly relevant given the widespread expression of EGFR in normal human tissues (e.g., skin and gastrointestinal tract), which poses risks of toxicity [41–43]. However, recent clinical studies demonstrating that EGFR-targeted CAR-T cells were well-tolerated in GBM patients provide preliminary evidence supporting the safety of localized EGFR targeting [6, 26, 30, 44].

Finally, while we have validated B7-H3 and EGFR knockout efficiency in vitro (Fig. S7) and demonstrated heterogeneous antigen expression in our mixed models (Fig. S11), we acknowledge that these models do not fully capture the spectrum of antigen heterogeneity observed in patient tumors. Future studies should incorporate patient-derived xenografts with naturally occurring heterogeneous antigen expression and explore strategies to prospectively identify patients most likely to benefit from this approach based on baseline antigen co-expression patterns.

The EGF/EGFR signaling system is highly conserved across species, and while its role in the rodent brain is well-established, its relevance in the adult human brain remains incompletely understood [45]. Future studies should focus on: (1) developing models that better recapitulate the human immune microenvironment, such as humanized mouse models; (2) exploring additional target combinations based on comprehensive antigen profiling of primary GBM specimens; and (3) optimizing CAR and BsTe designs to maximize efficacy while minimizing toxicity.

In summary, our study indicates that B7-H3-CAR-T cells engineered to secrete EGFR-BsTe can simultaneously engage tumor antigens via the CAR and through secreted bispecific T-cell engagers, resulting in enhanced antitumor activity in preclinical models. These findings support further investigation of B7-H3-CAR-T-EGFR-BsTe in patients with GBM, a population with high unmet medical need. CAR-T cells capable of secreting BsTes represent a promising strategy to overcome antigen heterogeneity in GBM, potentially paving the way for more effective T cell–based therapies.

## Methods

### Patient samples

Fresh peripheral blood mononuclear cells (PBMCs) and tumor tissues from GBM patients (n = 4; Supplementary Tables 1) were collected after informed consent (Mianyang Central Hospital Ethics Committee).

### Mice and cell lines

NSG mice were purchased from the Model Animal Resource Information Platform of Nanjing University, China. Mice were bred under pathogen-free conditions at the Institutional Animal Care and Use Committee of Sichuan University.

The human glioma cell lines U87 (RRID: CVCL_UR33), A172 (RRID: CVCL_0131), and T98G (RRID: CVCL_0556), as well as the viral packaging cell line HEK293T (RRID: CVCL_0063), were obtained from the American Type Culture Collection and maintained under conditions specified by the supplier. U251 (RRID: CVCL_0021) glioma cell line was originally purchased from Procell. GBM06 and GBM08 were isolated from recurrent GBM patients. All cell lines have been authenticated using STR profiling within the last 3 years. All experiments were performed with mycoplasma-free cells.

For certain experiments, CRISPR/Cas9 delivered via lentiviral vectors was used to disrupt the EGFR or B7-H3 genes. We designed and analyzed single guide RNAs (sgRNAs) targeting the *EGFR* and *B7-H3* genes using CHOPCHOP. The designed sgRNAs (*EGFR*: TGAGCTTGTTACTCGTGCCT, *B7-H3*: CTGGTGCACAGTTTCACCGA) were cloned into the lentiCRISPRv2-puro vector respectively. When specified, cell lines were infected with the pLenti-CMV-luc2-IRES-Puro virus to introduce click beetle green luciferase, followed by sorting on a BD FACSAria to obtain a population with uniform (100%) luciferase expression.

### Glioblastoma organoids (GBOs)

The tumor pieces from resected glioblastoma tissue of patients were distributed into ultra-low attachment 6-well culture plates (#4440, Corning) containing a specific GBO medium, as previously described [39]. In brief, the GBO medium comprises 50% DMEM:F12 (#11,320,033, Thermo Fisher Scientific), 50% Neurobasal (#21,103,049, Thermo Fisher Scientific), 1X N2 supplement (#17,502,001, Thermo Fisher Scientific), 1X B27 w/o vitamin A supplement (#12,587,001, Thermo Fisher Scientific), 1X NEAAs (#11,140,050, Thermo Fisher Scientific), 1X GlutaMax (#35,050,061, Thermo Fisher Scientific), 1X PenStrep (#15,140,122, Thermo Fisher Scientific), 1X 2-mercaptoethanol (#21,985,023, Thermo Fisher Scientific), and 2.5 μg/ml human insulin (#PHR8925, Sigma). These plates were then placed on an orbital shaker set to rotate at 120 rpm, within a sterile incubator maintained at 37 °C, with an atmosphere of 5% CO2 and 90% humidity. Organoid passage cultures were routinely cut into pieces with a diameter of ~200 μm using fine dissection scissors to prevent substantial necrosis in the center due to limited nutrient and oxygen diffusion.

### Generation of CAR constructs

The transgene cassettes for CD19-CAR, CD19-BsTe, B7-H3-CAR, and EGFR-BsTe were synthesized and subcloned into a second‑generation lentiviral backbone (Addgene #135,992) regulated by a human EF‑1α promoter. All CARs contained CD8 transmembrane, 4‑1BB, and CD3ζ domains. BsTe sequences were preceded by an Igκ leader and followed by a His‑tag. The VHH against B7-H3 were derived from our lab (patent number: CN202310929518.2). We sourced the anti‑EGFR and anti‑CD19 scFv sequences from the publicly accessible Cetuximab and blinatumomab clones, respectively.

### Production of CAR-T cells

Lentiviruses were generated by transfecting 293 T cells with the plasmid encoding the CAR-BsTe constructs, a plasmid encoding a VSV-G envelope (pMD2.G), and an empty backbone plasmid (psPAX2) using polyethyleneimine (PEI) Reagent (#408,727, Sigma-Aldrich) as previously described [46].

PBMCs were isolated from patients or healthy donors by Ficoll, and T cells were enriched (EasySep Human T Cell Enrichment Kit; #19,051, StemCell Technologies) and activated with T‑Cell TransAct (#130–111–160, Miltenyi Biotec). Cells were cultured in X‑VIVO 15 medium (#02-060Q, Lonza) with 8% FBS and 20 U/mL IL‑2 (#200–02-50UG, PeproTech) for 3 days.

The transduction of T cells was performed as previously described [46]. Briefly, retronectin‑coated plates (#T100AC, Takara Bio; 20 μg/ml) were loaded with lentivirus and centrifuged (2000 × g, 90 min). Activated T cells (2 × 10^5^) were added and spinoculated (400 × g, 30 min, 30 °C). Medium was supplemented with IL‑7 (#200–07-10UG, PeproTech; 10 ng/mL) and IL‑15 (#200–15-10UG, PeproTech; 5 ng/mL) every 2–3 days. Untransduced T cells (UTD) served as controls. CAR expression was detected by flow cytometry using FITC‑labeled recombinant B7‑H3 or CD19 protein.

### Flow cytometry

Antibodies for antigen detection: FITC anti-human EGFR (#386,306, Biolegend, RRID: AB_3675105), FITC isotype control antibody (#400,110, Biolegend, RRID: AB_2861401), PE anti-human B7-H3 (#351,004, Biolegend, RRID: AB_10720987), PE isotype control antibody (#400,114, Biolegend, RRID: AB_326435), APC anti-human IL13RA2 (#MA5-40,955, Invitrogen, RRID: AB_2898716), APC isotype control antibody (#Ab232814, Abcam). For phenotyping: BV510 anti-human CD45 (#368,526, Biolegend, RRID: AB_2687377), Percp-cy5.5 anti-human CD3 (#981,008, Biolegend), PE-CY7 anti-human CD45RO (#304,230, Biolegend, RRID: AB_11203900), FITC anti-human CD62L (#980,702, Biolegend), APC anti-human PD1 (#379,208, Biolegend, RRID: AB_2922606), BV421 anti-human TIM3 (#364,808, Biolegend, RRID: AB_3068174), PE anti-human LAG3 (#369,306, Biolegend, RRID: AB_2629592). Dead cells were excluded via a Zombie NIR™ viability dye (Biolegend #423,106) before applying an Fc receptor blocking reagent (Biolegend #422,302, RRID: AB_2818986). Subsequently, the cells were stained with respective antibodies at room temperature for 15 min in the dark. After washing with PBS, the cells were analyzed on a CytoFLEX (Beckman Coulter). The analysis was conducted using FlowJo V.10.5.3.

### Co-culture repeat stimulation assay

CAR‑T or UTD cells were cocultured with tumor cells (2:1 ratio) in duplicate 24‑well plates. One plate was used for counting and phenotyping every 3 days; the other for the next stimulation round. T cells (CD45⁺) and tumor cells (CD45⁻) were quantified by flow cytometry after Zombie NIR staining. T-cell expansion was assessed by counting the number of T cells and comparing it to the initial number of T cells added to the culture on day 0. The percentage of viable tumor cells was determined by counting the viable tumor cells and comparing this number to the number of tumor cells in the control group (which was not treated with T cells).

### CFSE Cell proliferation assay

T cells were labeled with CFSE Cell Division Tracker Kit (#423,801, BioLegend), then cultured with GBM06 cells (E/T = 2:1) for 6 days. Cells were stained with Zombie NIR and BV605 anti‑CD3 (#317,321, BioLegend, RRID: AB_11126166), and analyzed by flow cytometry.

### Cytotoxicity assay

Luciferase‑labeled target cells were co‑incubated for 18 h with either CAR‑T cells or UTD at the specified E/T ratios. For evaluation of secreted factors, UTD T cells were combined with 100 μL of CAR‑T supernatant. At the end of the incubation, a luciferase assay kit (#E1500, Promega) was used to lyse the cells, and luminescence was quantified on a BioTek Synergy H4 hybrid reader. Specific lysis percentages were derived from the equation: (control counts − experimental counts)/control counts ×100%.

### Real-time cytotoxicity assay

Cytotoxic activity was assessed using the xCELLigence Real-Time Cell Analysis (RTCA) system (ACEA Biosciences). Briefly, target cells were seeded in a 96-well E-plate, equilibrated within the instrument, and background measurements were recorded. Following a 30-min room temperature incubation, plates were returned to the RTCA station for overnight attachment. Subsequently, each well received effector cells, and the impedance (recorded as the cell index) was measured at 30‑minute intervals throughout the experiment.

### ELISA

Effector cells were cocultured with tumor cells (1:1) for 18 h. Supernatants were assayed for Perforin (#EHC154.96, NeoBioscience Technology), Granzyme B (#EHC117.96, NeoBioscience Technology), IFN‑γ (#1,110,002, Dakewe Biotech), and TNF‑α (#1,117,202, Dakewe Biotech) using commercial ELISA kits following the manufacturer’s protocols. The absorbance values were measured using a Synergy H4 Hybrid microplate reader (BioTek, USA).

### Western blot analysis

To detect secreted bispecific T-cell engagers (BsTe), supernatants from CAR-T cell cultures were collected and concentrated using ultrafiltration membranes with a 10,000 Dalton molecular weight cut-off (#UFC901024, Millipore). Equal volumes of concentrated supernatants were resolved by SDS-PAGE and transferred onto nitrocellulose membranes (GE Healthcare). After blocking, membranes were treated with a monoclonal anti‑His‑Tag antibody (Proteintech, #66,005‑1‑Ig) in TBST/5% non‑fat dry milk for 1 h (25 °C) and then overnight at 4 °C. After three washes with TBST, membranes were incubated with an HRP-conjugated anti-mouse secondary antibody (#SA00001-1, Proteintech) in TBST for 1 h at 25 °C, and then washed three additional times with TBST. Protein signals were visualized using the e-BLOT Touch Imager (e-BLOT).

### GBO coculture assay

Spherical GBOs with diameters ranging from 200 to 500 μm were manually selected and cocultured in ultra-low attachment 24-well culture plates (#4441, Corning) with various sets of CAR-T cells at an approximate E/T ratio of 1:1. The cell counts of GBOs were determined by digesting similarly sized GBOs from each patient with StemPro™ Accutase™ (#A1110501, Thermo Fisher Scientific) and then counting the cells using an Automated Cell Counter (Countstar). The GBOs were harvested 2 days following CAR-T cell treatment.

### Immunofuorescence

The following antibodies were used in the immunofuorescence staining. For direct staining biomarkers of GBOs: FITC anti-human CD133 (#397,908, Biolegend, RRID: AB_3068114), Alexa Fluor 594 anti-human NESTIN (#656,804, Biolegend, RRID: AB_2563509), and Alexa Fluor 647 anti-human SOX2 (#656,108, Biolegend, RRID: AB_2563681). For detecting the expression of EGFR or B7-H3 or IL13RA2 on GBOs: FITC anti-human EGFR (#386,306, Biolegend, RRID: AB_3675105), PE anti-human B7-H3 (#351,004, Biolegend, RRID: AB_10720987), APC anti-human IL13RA2 (#MA5-40,955, Invitrogen, RRID: AB_2898716). For detecting the infiltration of CAR-T cells in the GBO coculture assay: anti-CD3 (#85061S, Cell Signaling Technology, CST), AF488-labeled Goat Anti-Rabbit IgG (#A0423, Beyotime Biotechnology). Samples were fixed, permeabilized, and counterstained with DAPI. The z-stack images were obtained via an Olympus SpinSR10 instrument equipped with 10 × or 20 × objective lenses and were analyzed utilizing the Olympus OlyVIA analysis software.

### Immunohistochemistry

Specimens fixed in formalin and embedded in paraffin originated either from experimental tumors or from resected GBM patient tissues. The tumor slices were sectioned at a thickness of 5 μm and stained using commercial monoclonal antibodies against EGFR (#4267L, CST), B7-H3 (#14058L, CST), and CD3 (#85061S, CST), all diluted to a ratio of 1:200, following antigen retrieval with EDTA, and in accordance with the manufacturer's standard protocols. Hematoxylin and eosin (H&E) staining was conducted by the Lilai Biomedicine Experiment Center (Chengdu, Sichuan). Subsequently, the stained slides were mounted and scanned using a Pannoramic MIDI scanner (3DHISTECH).

### Multiplex immunohistochemistry

Formalin-fixed, paraffin-embedded 5 µm tissue sections from the experimental mice were stained using the IRISKit HyperView multiplex immunostaining kit (Catalog Nos. MH900206) following the manufacturer's instructions. The antibodies utilized were rabbit monoclonal EGFR (#4267L, CST), rabbit monoclonal B7-H3 (#14058L, CST), and rabbit monoclonal IL13RA2 (#85677S, CST). The slides were imaged with an Olympus VS200 slide scanner.

### In vivo treatments

All animal procedures were reviewed and approved by the West China Hospital of Sichuan University Biomedical Ethics Committee (ethical approval document: 20,230,426,002). NSG mice, aged 6 to 8 weeks, were anesthetized using ketamine/xylazine (90/10 mg/kg) and underwent stereotactic injection with 2 × 10^5^ GBM06 cells into the right frontal lobe of the brain (2 mm lateral and 1 mm anterior to bregma, at a depth of 3 mm). Tumor progression was assessed by luminescence emission using a Clinx IVIS Spectrum, following intraperitoneal injection of D-luciferin (#ST196, Beyotime Biotechnology) as per the manufacturer's instructions. CAR-T cells were thawed, washed twice with PBS, and resuspended to a concentration of 5 × 10^6^ CAR-T cells per 100 μL of PBS before being injected intravenously through the tail vein. Body weight was monitored twice weekly. Survival was tracked over time until the mice became moribund, exhibiting neurological impairments and significant weight loss.

### In vivo BsTe biodistribution

On day 18 after two CAR‑T infusions, GBM06 tumor‑bearing mice were sacrificed. Tissue samples (tumor, heart, liver, spleen, lung, kidney, brain) were collected, diced into 5 mm^3^ pieces, and snap‑frozen or set aside for IHC. Frozen tissues were homogenized in RIPA buffer (Beyotime #P0013B) with 1 mM PMSF (Beyotime #ST506), rotated at 4 °C for 30 min, and centrifuged at 14,000 × g (4 °C, 10 min). Supernatants were analyzed for protein content (BCA kit, Beyotime #P0010) and BsTe levels (ELISA). BsTe concentrations are expressed as picograms per milligram of total protein. IHC assessed human CD3⁺ T‑cell infiltration.

### Statistics

Statistical analysis was conducted with GraphPad Prism 9. Student’s t‑test was used for unpaired two‑group comparisons. One‑way ANOVA followed by Tukey’s test served for multiple groups with a single independent variable, whereas two‑way ANOVA with Šidák’s test was applied when two or more independent variables were involved. Median survival was calculated by the Kaplan‑Meier method, and survival curves were compared using the log‑rank test. All data are expressed as mean ± SD, with *P* < 0.05 considered significant.

## Supplementary Information

Supplementary Material 1.

## Data Availability

All data relevant to the study are included in the article or uploaded as supplementary information.

## Abbreviations

B7-H3: B7 homolog 3 protein
BsTe: Bispecific T-cell engager
CAR: Chimeric antigen receptor
CFSE: Carboxyfluorescein succinimidyl ester
EGFR: Epidermal growth factor receptor
ELISA: Enzyme-linked immunosorbent assay
GBOs: Glioblastoma organoids
GBM: Glioblastoma
LGG: Low-grade glioma
mAbs: Monoclonal antibodies
OS: Overall survival
PBMC: Peripheral blood mononuclear cell
RTCA: Real-Time Cell Analysis
RNA-seq: RNA sequencing
TMZ: Temozolomide
TME: Tumor microenvironment
UTD: Untransduced T cells
