# Supplementary material for: B7 homolog 3-targeted CAR-T cells secreting EGFR T-cell engagers for improved control of glioblastoma progression
Source: Mol Biomed. 2026 Jun 16;7:92. doi: 10.1186/s43556-026-00492-7 (PMC13272847; doi:10.1186/s43556-026-00492-7)
Supplement: Supplementary file 1 — Supplementary Material 1. [file 43556_2026_492_MOESM1_ESM.pdf]

Supplementary information

The supplementary files are provided to assist in understanding the research: B7 homolog 3-targeted CAR-T cells secreting EGFR T-cell engagers for improved control of glioblastoma progression.

Zongliang Zhang[1#], Nian Yang[1#], Hui Zeng[2#], Yongdong Chen[1], Huaqing Lu[1], Long Xu[1], Zeng Wang[1], Guoqing Wang[*3], Liangxue Zhou[*2,4], Aiping Tong[*1,5]

[1]State Key Laboratory of Biotherapy and Cancer Center, Research Unit of Gene and Immunotherapy, Chinese Academy of Medical Sciences, Collaborative Innovation Center of Biotherapy, West China Hospital, Sichuan University, Chengdu Sichuan Province, 610041 China.

[2]Department of Neurosurgery, Mianyang Central Hospital, Mianyang, Sichuan, 621000, China.

[3]Department of Ophthalmology, West China Hospital, Sichuan University, West China Medical School, Chengdu, Sichuan, 610041, China.

[4]Department of Neurosurgery, West China Hospital, West China Medical School, Sichuan University, Chengdu, Sichuan, 610041, China.

[5]Frontiers Medical Center, Tianfu Jincheng Laboratory, Chengdu 610212, China.

[#]These authors contributed equally to the manuscript.

[*]Corresponding Author:

Dr Aiping Tong; aipingtong@scu.edu.cn & Dr Liangxue Zhou; zhlxlll@163.com& Dr Guoqing Wang; sivanwgq@gmail.com

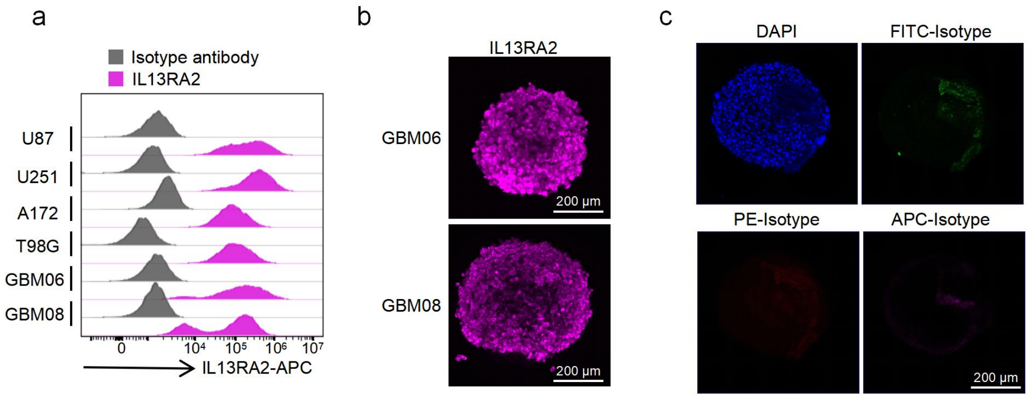

Figure S1. IL13RA2 expression in GBM cell lines and patient-derived organoids. (a) Flow cytometric analysis of IL13RA2 expression in the indicated GBM cell lines (U87, U251, A172, T98G, GBM06, and GBM08). Gray histograms represent isotype controls; colored histograms represent IL13RA2 staining. (b) Representative immunofluorescence images of IL13RA2 staining in organoids derived from GBM06 and GBM08 patient tumors. Scale bars: 200 µm. (c) Isotype antibody control staining for the immunofluorescence experiments shown in (Fig. 1b). Scale bars: 200 µm.

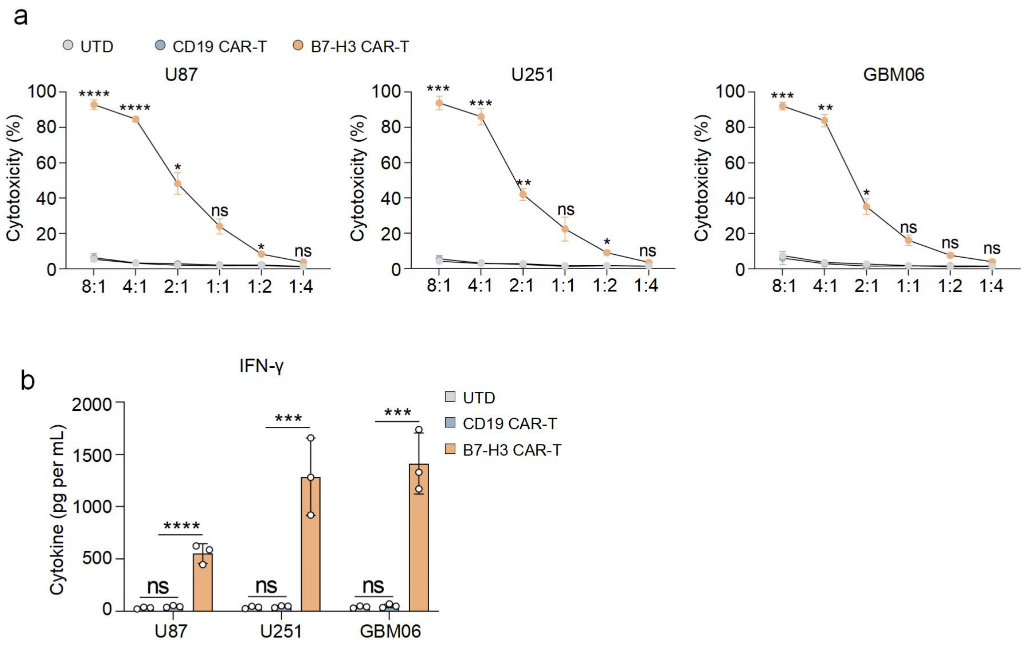

Figure S2. Cytotoxicity analysis of B7-H3 CAR-T cells. (a) Cytolytic activity of B7-H3 CAR-T and CD19 CAR-T against U87, U251, and GBM06 cells, at various effector-to-target (E/T) ratios for 24 hours incubation. Values represent mean ± SD, n = 3. *$P < 0.05$; **$P < 0.01$; ***$P < 0.001$; ****$P < 0.0001$; ns, not significant. (b) IFN-γ cytokine release upon co-culture with U87, U251, and GBM06 cells at a 1:1 E/T ratio for 24 hours of incubation. Values represent the mean ± SD, n = 3. ***$P < 0.001$; ****$P < 0.0001$; ns, not significant.

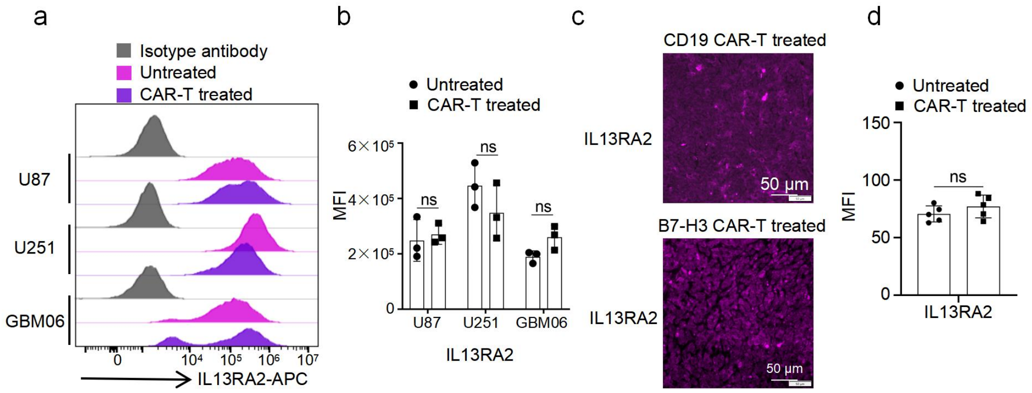

Figure S3. IL13RA2 expression is maintained in GBM cells following B7-H3 CAR-T cell treatment. (a) Representative flow cytometry histograms showing IL13RA2 expression on residual GBM cells after co-incubation with B7-H3 CAR-T cells, compared to untreated controls. (b) Quantification of IL13RA2 mean fluorescence intensity (MFI) in untreated versus B7-H3 CAR-T-treated groups. Data are presented as mean ± SD from three independent experiments. Statistical analysis was performed using two-way ANOVA with Šídák's multiple comparisons test; ns, not significant. (c) Multiplex immunohistochemistry (mIHC) staining for IL13RA2 on coronal brain sections from mice bearing GBM xenografts treated with CD19 CAR-T or B7-H3 CAR-T cells. Representative images are shown. (d) Quantification of IL13RA2 MFI from the multiplex IHC staining in (c). Data are presented as mean ± SD (n = 5 mice per group). Statistical significance was determined by unpaired two-tailed Student's t-test; ns, not significant.

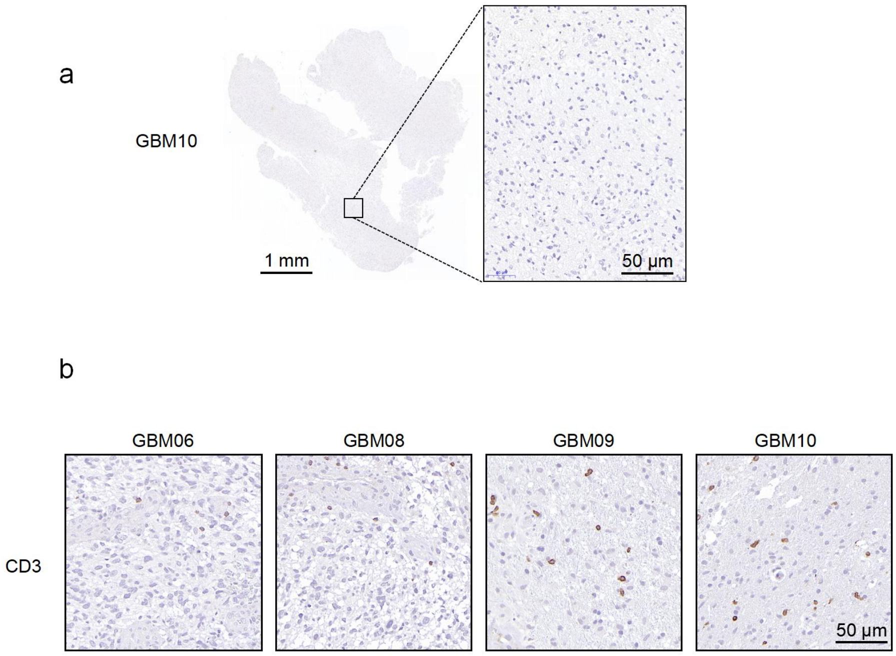

Figure S4. Immunohistochemical analysis of GBM patient tissues. (a) Representative images of negative control staining for IHC using isotype control antibodies. Scale bars: 1 mm (left panel), 50 µm (right panel). (b) Detection of tumor-infiltrating T cells in four patient-derived GBM tissues by IHC using an anti-CD3 antibody. Brown staining indicates CD3-positive T cells. Scale bars: 50 µm.

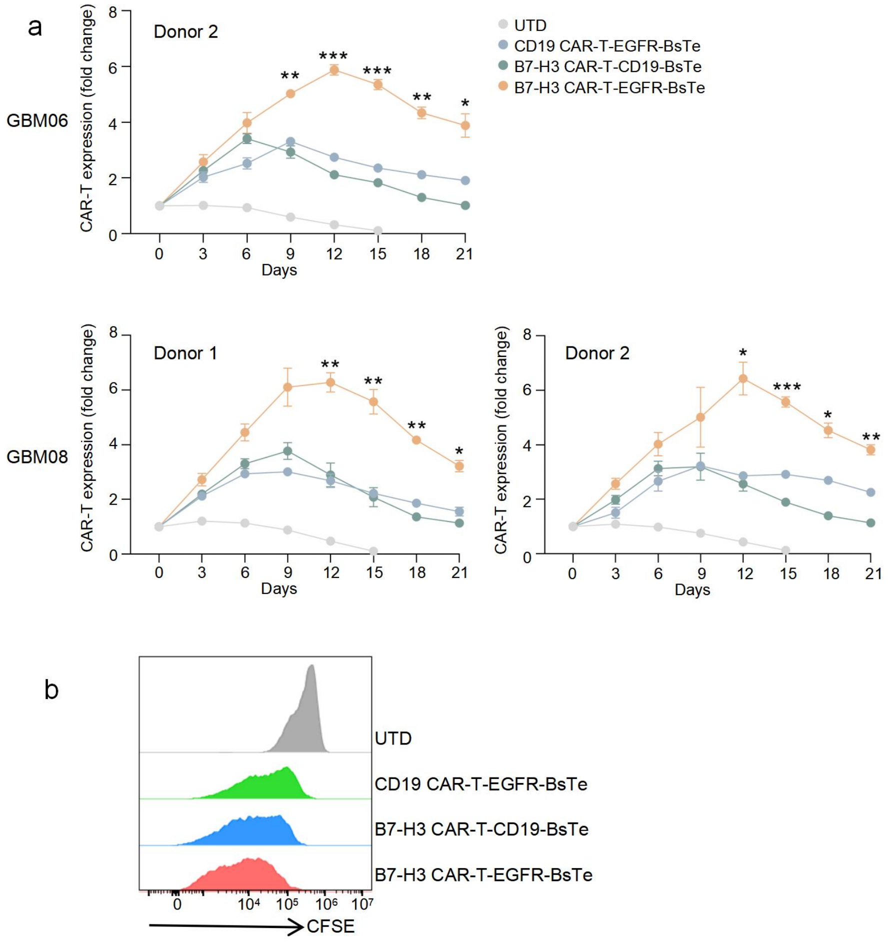

Figure S5. Proliferation and expansion of CAR-T cells upon repeated antigen stimulation. (a) CAR-T cells generated from two independent healthy donors were stimulated by repeated co-culture with GBM06 and GBM08 cells over a 21-day period. Tumor cells were refreshed every three days, and CAR-T cell expansion was monitored at each round of the serial co-culture assay. Data are presented as mean ± SD of triplicate wells. Statistical analysis was performed using two-way ANOVA with Šídák's multiple comparisons test. *P < 0.05; **P < 0.01; ***P < 0.001. (b) Proliferative capacity of CAR-T cells was assessed by CFSE dilution. T cells were labeled with CFSE prior to the initiation of serial co-culture, harvested on day 6, and analyzed by flow cytometry. Representative histograms and quantification are shown.

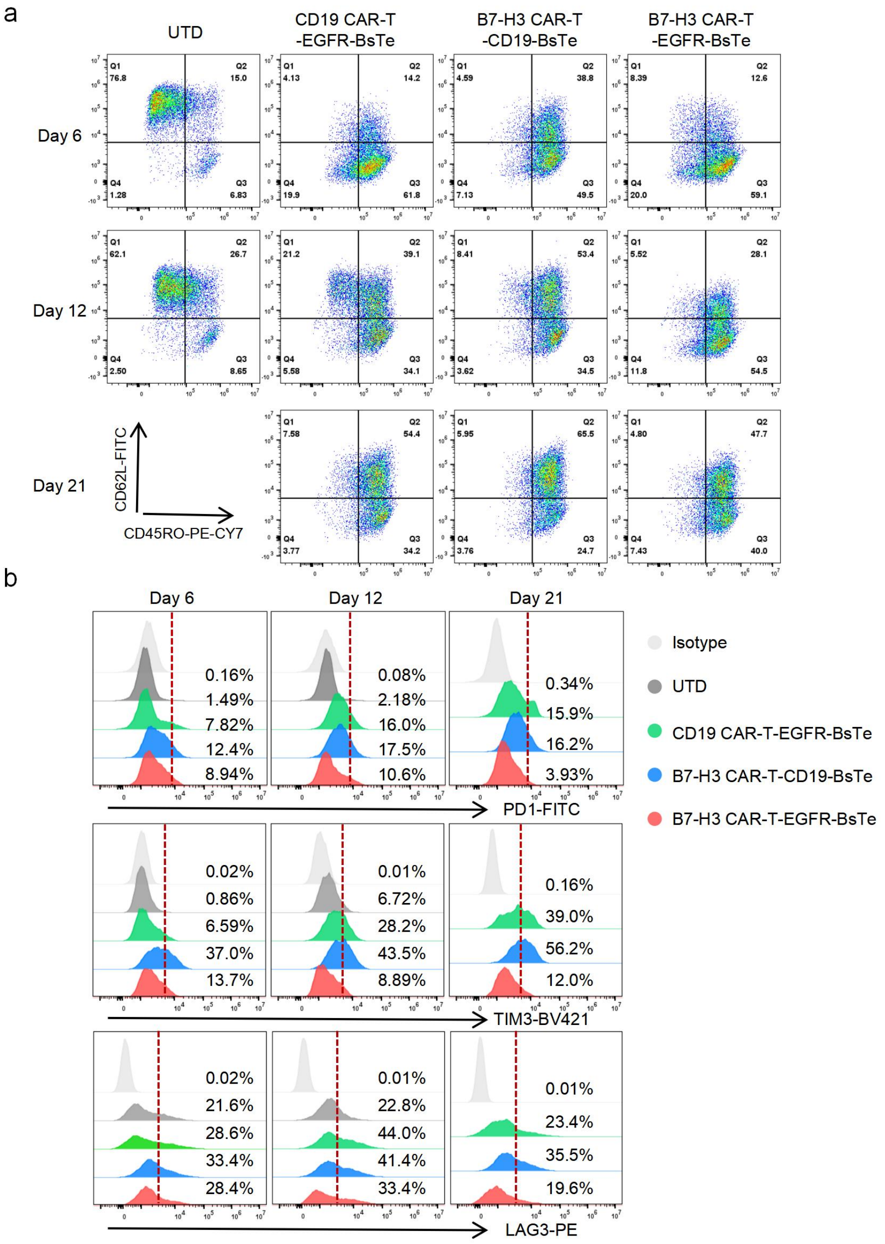

Figure S6. Phenotypic characterization and exhaustion marker expression of CAR-T cells during repeated stimulation. CAR-T cells were co-cultured with GBM target cells in a repeated stimulation assay as described in Figure 3. Flow cytometric analysis was performed on days 6, 12, and 21 to assess T-cell differentiation and exhaustion status. Representative flow cytometry plots are shown. (a) T-cell phenotype was evaluated by examining the expression of CD45RO and CD62L to distinguish naive ($CD45RO^{-}CD62L^{+}$), effector memory ($CD45RO^{+}CD62L^{-}$), and effector ($CD45RO^{-}CD62L^{-}$) T-cell subsets.(b) T-cell exhaustion was assessed by monitoring the expression of inhibitory receptors TIM-3, PD-1, and LAG-3 on $CD3^{+}$ T cells.

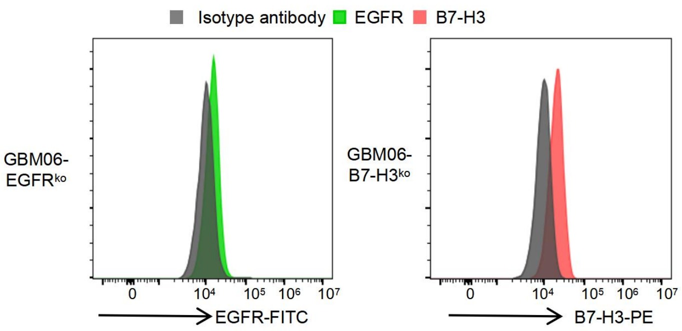

Figure S7. B7-H3 and EGFR expression were detected in GBM06-B7-H3^{ko} and GBM06-EGFR^{ko} cell lines. Flow cytometry was used to confirm the B7-H3 and EGFR knockout efficiency.

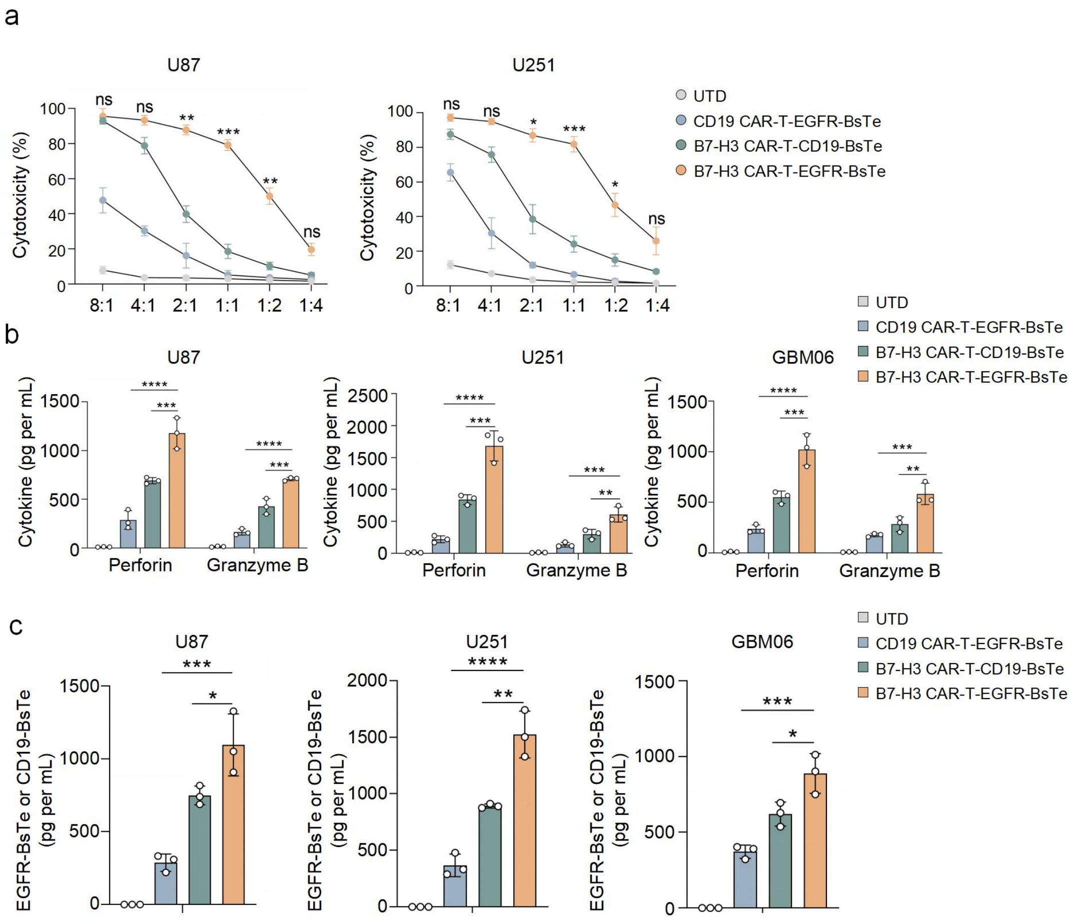

Figure S8. B7-H3-CAR-T-EGFR-BsTe cells demonstrate enhanced cytotoxicity and effector molecule release in vitro. (a) Cytotoxicity of the indicated CAR-T cells against U87 and U251 glioma cell lines was assessed using a bioluminescence-based assay after 18 hours of co-culture at various E:T ratios. Data are presented as mean±SD from three independent donors. Statistical comparisons between the B7-H3-CAR-T-CD19-BsTe and B7-H3-CAR-T-EGFR-BsTe groups were performed using two-way ANOVA with Šídák's multiple comparisons test. *P < 0.05; **P < 0.01; ***P < 0.001; ns, not significant. (b) Cytokine release from CAR-T cells. UTD or CAR-T cells were co-cultured with the indicated GBM cell lines (U87, U251, or GBM06) at a 1:1 E:T ratio for 18 hours. The concentrations of Perforin and Granzyme B in the supernatants were measured by ELISA. Data are shown as mean ± SD from three technical replicates and are representative of three independent experiments. Statistical significance was determined by two-way ANOVA with Tukey's multiple comparisons test. **P < 0.01; ***P < 0.001; ****P < 0.0001. (c) Quantification of BsTe secretion. BsTe concentrations in the post-co-culture supernatants from the experiments described in (b) were measured by ELISA to confirm bioactivity. Data are representative of three independent experiments and were analyzed by one-way ANOVA. *P < 0.05; **P < 0.01; ***P < 0.001; ****P < 0.0001.

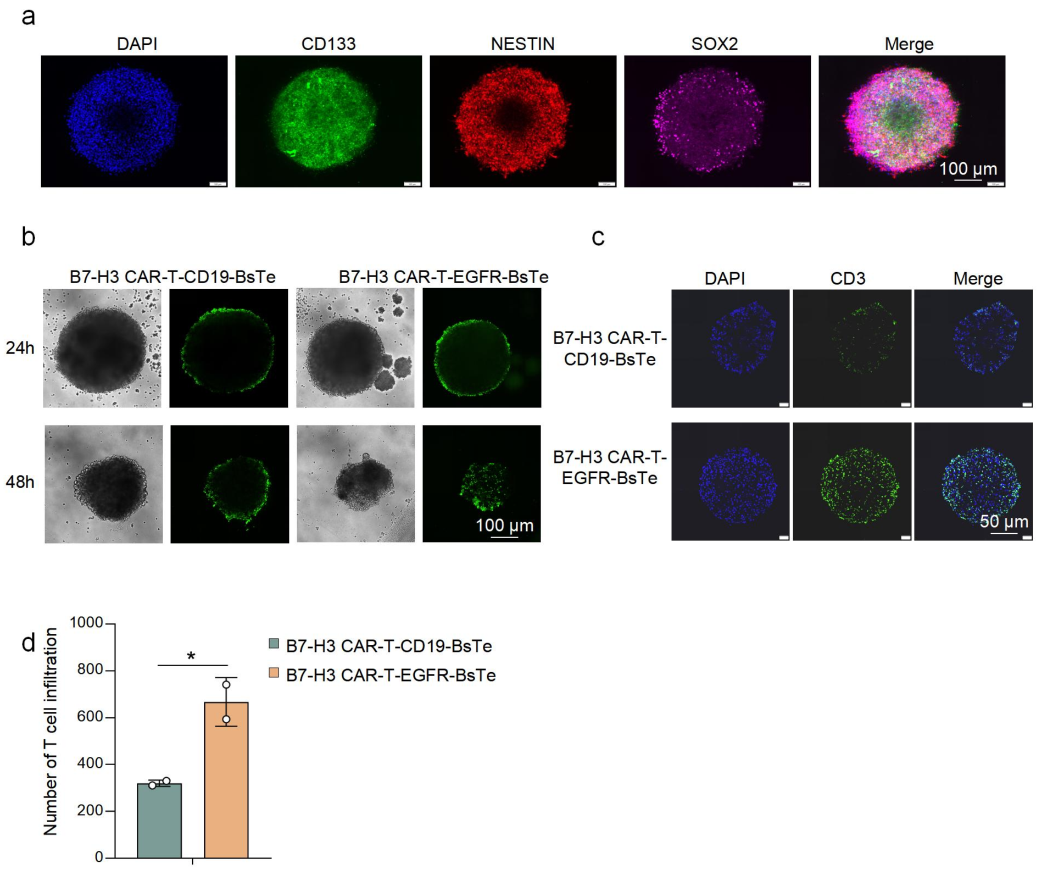

Figure S9. Characterization of GBM organoids and CAR-T cell infiltration (related to Figure 4f and 4g). (a) Representative immunofluorescence images of GBM08 patient-derived organoids stained for stem/progenitor markers CD133 (green), NESTIN (red), and SOX2 (magenta). Nuclei were counterstained with DAPI (blue). Scale bar: 50 μm. (b) B7-H3-CAR-T-CD19-BsTe and B7-H3-CAR-T-EGFR-BsTe cells were pre-labeled with CFSE (green) and co-cultured with GBM08 organoids for 24 or 48 hours. Representative confocal images show T-cell accumulation and infiltration at the indicated time points. Scale bars: 100 μm. (c) Following the co-culture described in (b), GBM08 organoids were fixed with paraformaldehyde, paraffin-embedded, and sectioned. Immunofluorescence staining for CD3 (green) was performed to visualize infiltrating T cells. Nuclei were counterstained with DAPI (blue). Scale bars: 50 μm. (d) Quantification of CD3-positive T cells within GBM06 and GBM08 organoids from the experiments shown in (c) and Figure 4h. Data are presented as mean ± SD. Statistical analysis was performed using unpaired t-test; *P < 0.05; **P < 0.01; ***P < 0.001. All images were acquired using an Olympus SpinSR10 confocal microscope.

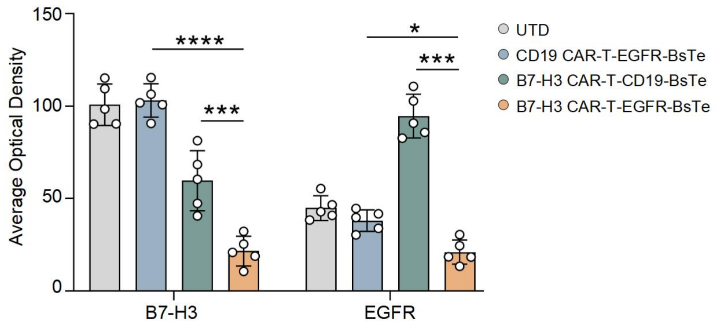

Figure S10. Quantification of B7-H3 and EGFR immunohistochemistry staining in mouse brain sections (related to Figure 5f). Quantification of B7-H3 and EGFR expression in coronal brain sections from mice bearing GBM xenografts treated with the indicated CAR-T cell groups (UTD, CD19-CAR-T-EGFR-BsTe, B7-H3-CAR-T-CD19-BsTe, and B7-H3-CAR-T-EGFR-BsTe). Significant downregulation of B7-H3 was observed in both B7-H3 CAR-T cell-treated groups compared to controls. Marked upregulation of EGFR was detected in tumors from mice treated with B7-H3-CAR-T-CD19-BsTe cells (consistent with Fig. 1e). Data are presented as mean ± SD (n = 5). Statistical analysis was performed using one-way ANOVA with Dunnett's multiple comparisons test; *P < 0.05; ***P < 0.001; ****P < 0.0001.

a

b

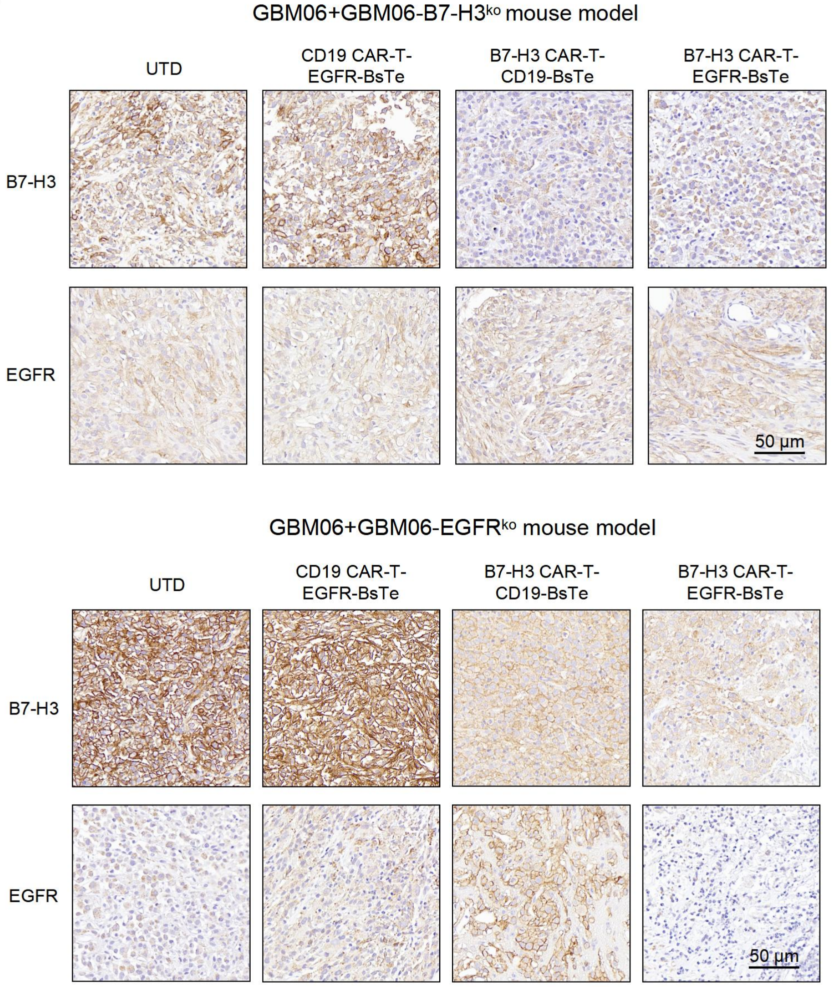

Figure S11. Immunohistochemical analysis of B7-H3 and EGFR expression in mixed tumor models (related to Figure 6). (a) IHC staining and quantification of B7-H3 and EGFR expression in the GBM06 + GBM06-B7-H3^{ko} mixed tumor model. Significant downregulation of B7-H3 was observed in tumors from both B7-H3 CAR-T cell-treated groups, with no significant difference between B7-H3-CAR-T-CD19-BsTe and B7-H3-CAR-T-EGFR-BsTe treatment. EGFR expression was comparable across all treatment groups, and no upregulation of EGFR was detected in B7-H3-CAR-T-CD19-BsTe-treated tumors in this mixed model. Representative images are shown. Scale bars, 50 μm. (b) IHC staining of B7-H3 and EGFR in the GBM06 + GBM06-EGFR^{ko} mixed tumor model. Tumors from mice treated with B7-H3-CAR-T-EGFR-BsTe cells exhibited more pronounced downregulation of B7-H3 compared to those receiving control CAR-T cells. In contrast, tumors from B7-H3-CAR-T-CD19-BsTe-treated mice showed marked upregulation of EGFR, consistent with our previous observations (Fig. 1e and Fig. 5f), whereas this upregulation was abrogated in the B7-H3-CAR-T-EGFR-BsTe-treated group. Representative images are shown. Scale bars, 50 μm.

Supplementary Table 1. GBM patients with clinical features

Clinical characteristics	GBM patient NO.1 (GBM06)	GBM patient NO.2 (GBM08)	GBM patient NO.3 (GBM09)	GBM patient NO.4(GBM10)
Age	patient in their 50s	patient in their 50s	patient in their 40s	patient in their 60s
Tumor location	Temporal	Frontal	Temporal	Frontal
KPS	90	90	80	90
Tumor size (cm)	4.1*3.2*4.4	6.2*4.9*4.7	6.9*3.8*4.8	5.2*3.8*3.9
WHO grade	WHO IV	WHO IV	WHO IV	WHO IV
